# Antibiotic resistance and virulence shape the population of *E. coli* from diseased calves in Wallonia, Belgium

**DOI:** 10.1038/s44259-026-00235-2

**Published:** 2026-06-25

**Authors:** Virginie Guérin, Nicolas Cabanel, Gan Min Marissa Diana Meijer, Guilhem Royer, Jean-Noël Duprez, Jacques G. Mainil, Marc Saulmont, Damien Thiry, Philippe Glaser

**Affiliations:** 1 Unité EERA, Institut Pasteur, Paris, France; 2 Université Paris-Cité, Paris, France; 3 Veterinary Bacteriology, Department of Infectious and Parasitic Diseases, FARAH and Faculty of Veterinary Medicine, ULiège, Liège, Belgium; 4 Regional Animal Health and Identification Association (ARSIA), Ciney, Belgium; 5 Present Address: École Nationale Vétérinaire d’Alfort, 7, Avenue du Général de Gaulle, Maisons-Alfort, France; 6 Present Address: Institute of Biology—Molecular Microbiology, Humboldt-Universität zu Berlin, Berlin, Germany

**Keywords:** Ecology, Ecology, Microbiology

## Abstract

*Escherichia coli* is a major cause of deadly infections in calves. As surveillance in livestock is mainly performed on carriage isolates, factors driving the dissemination of *E. coli* infecting calves remain largely unknown. Here, we analyzed the population of β-lactam-resistant *E. coli* in sick calves across five calving seasons on 444 farms in Wallonia, Belgium. 99% of the isolates were MDR and enriched in virulence factors, adhesins and toxins. Restrictions on critical antibiotic usage have no impact on population structure and β-lactamase gene content. This resilient population is likely shaped by virulence and resistance. Correlations of phylogeny and geographic origin suggest indirect local transmission with differences between regions east and west of the Meuse. Phylogenetic analyses with isolates from EnteroBase show a close relationship of calf isolates with human isolates, suggesting host jumps. These findings provide new means to further model the spread of *E. coli* in livestock farming.

## Introduction

*Escherichia coli* is a ubiquitous bacterium, colonizing the gut of humans and animals. It is primarily a commensal but also responsible for digestive and extra-intestinal infections, mainly urinary tract infections, sepsis and meningitis in humans and animals or mastitis in cattle^1,2^. In young calves, mainly in the first days of life, *E. coli* is one of the most frequent causes of diarrhea and septicemia^3^, associated with major economic losses^4^. This leads to heavy antibiotic use in calf farming^5^. The use of antibiotics in humans and animals contributes to the selection for antibiotic resistance and to the emergence and dissemination of multidrug-resistant (MDR) bacteria. The consequent increase of MDR *E. coli*, especially those expressing extended spectrum β-lactamase (ESBL) and their transmission between humans, animals, and the environment, represent a One Health threat^6^.

In 2005, the World Health Organization published a first ranking of medically important antimicrobials, which is regularly updated^7^. This provided guidelines for restricting the use of antibiotics in food-producing animals^8^, as a major share of antibiotics is used in this sector^9^. In Belgium, a Royal Decree, applied since 2016, restricts the use of antibiotics considered critical to human health, such as 3rd- and 4th- generation cephalosporins (3GC and 4GC) in production animals^10^.

To analyze the impact of this regulation, the β-lactam resistance profiles of 3917 *E. coli* isolates from 3537 sick calves were analyzed^11^. They were collected from 2014 to 2021 in Wallonia, Belgium. Analysis of the trends between the seven calving seasons showed a decrease in the two β-lactam resistance profiles associated with 3GC resistance, namely extended-spectrum β-lactamase (ESBL) and acquired cephalosporinase (AmpC). The reduction of resistance to 3GC was likely due to the decreased use of 3GC^11^. However, it was compensated by an increase in isolates resistant to amoxicillin but not to 3GC.

Antibiotic resistance among a bacterial species results from a combination of selection and transmission^12^. Transmission routes are difficult to track and require combining epidemiological and genomic data^13^. The spread of ESBL- and plasmid-encoded cephalosporinase-producing *E. coli* within and between the human, animal and environmental sectors has been recently reviewed^6^. Although recent studies based on WGS led to variable results in transmission, they revealed mainly different situations between high-income countries (HIC) and low- and medium-income countries (LMIC). In HIC, mainly intrasectoral (human, animal, or environmental) transmissions were predicted, but in LMIC, intersectoral transmissions were also prevalent. However, the factors driving the spatiotemporal dynamics of antibiotic-resistant *E. coli* strains in livestock across a large geographic area remain largely unknown.

Through the surveillance of β-lactam-resistant *E. coli* infecting calves, we have obtained a dense sampling of isolates from Wallonia^11,14^. Here, we characterized the temporal and geographic dynamics of *E. coli* in sick calves by sequencing 764 isolates from 444 farms across Wallonia over five calving seasons (2014 to 2020). Despite the reduction in the rate of 3GC resistance, we did not observe a significant change in the population structure or β-lactam resistance mechanisms across the three more deeply sampled calving seasons (2016–2020). Isolates were not only MDR but also enriched in virulence factors compared to EnteroBase^15^ isolates. It suggested that virulence and host adaptation contribute to the resilience of this population. By correlating genetic and geographical data, we quantified the rate of dissemination and observed different patterns depending on the different breeding regions. Furthermore, by including *E. coli* genome sequences from the international database EnteroBase in the phylogenetic analysis, we showed that highly resistant, locally disseminated lineages of calf isolates include isolates that infect humans, suggesting host jumps.

## Results

### Diversity of *E. coli* from sick calves isolated in Wallonia

**Fig. 1:**
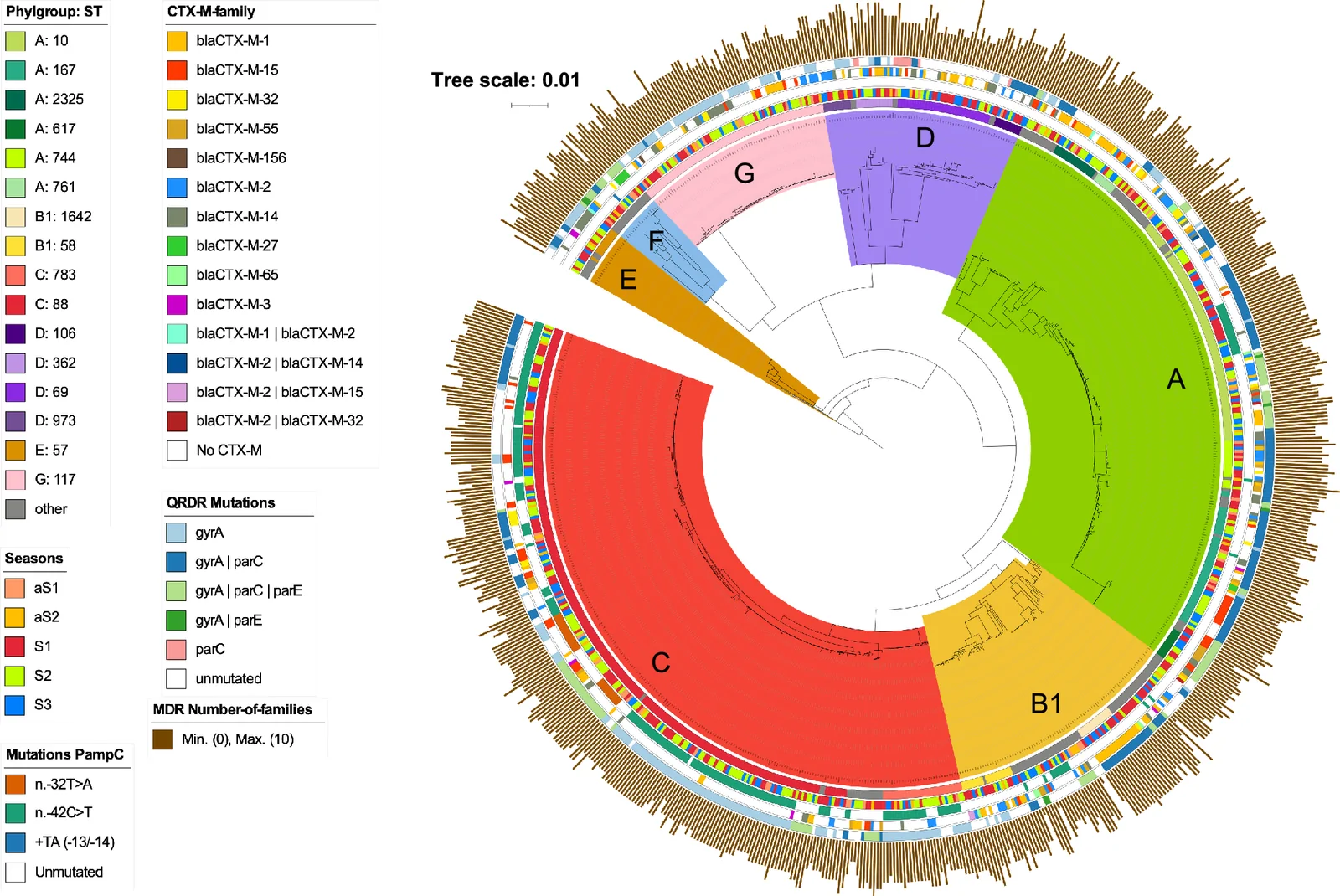
Phylogeny and characteristics of the 764 *E. coli* isolates from calves. Phylogeny was performed by maximum likelihood by using Roary, 3.13.0 [44] and RAxML 8.2.12^45^,with *Escherichia fergusonii* (NZ_CP057657) as outgroup. Genomes were annotated according to the figure key as circles from inside to outside: the main ST and the corresponding phylogroup, the collecting season, P_*ampC*_ mutations, *bla*_CTX-M_ genes, mutations in the *gyrA* and *parC* quinolone resistance determining regions (QRDR) and in *parE*, and the number of resistances to antibiotic families based on genes and mutations identified. Details are in Tables S1 and S2. The tree was visualized with iTOL v7^47^.

To analyze the temporal dynamics of the *E. coli* population in sick calves, we selected 681 isolates for WGS from 1495 ampicillin-resistant *E. coli* collected from 831 farms over three calving seasons (S1 to S3, November to February 2017 to 2020) (Table 1, Table S1, and Fig. S1)^11^. Isolates were classified into four β-lactam resistance profiles: NSBL (Narrow-Spectrum-β-lactamase, amoxicillin resistant only), ESBL (Extended-Spectrum-β-lactamase, 3GC resistant with clavulanic acid synergy), AmpC (Cephalosporinase, 3GC and 4GC resistant without clavulanic acid synergy), and AmpC-like (amoxicillin resistant with no or reduced clavulanic synergy and cefoxitin resistant) as further described in the Methods section^11^. Isolates were chosen to keep the balance between the four β-lactam resistance profiles across the three calving seasons (Table 1). They came from 379 farms spread across Wallonia. We added 83 isolates from the two previous calving seasons (aS1 and aS2, November to February 2015 to 2017, Table S2) to increase the genomic diversity over a longer period. We evaluated the diversity of these 764 isolates by performing core genome phylogeny (Fig. 1). Despite their isolation from sick animals, all phylogroups (A, B1, C, D, E, F, and G)^16^ were observed, except B2, which is usually associated with extraintestinal infections in humans^1^.

**Table 1:** Collected and sequenced *E. coli* isolates according to the three calving seasons (S1-3) and the β-lactam resistance profiles^a^

^b^β-lactam resistance profiles	Pathogenic						Non-pathogenic						Total WGS/collected
	S1 (2017–2018)		S2 (2018–2019)		S3 (2019–2020)		S1 (2017–2018)		S2 (2018–2019)		S3 (2019–2020)		
	^c^WGS	Collected	WGS	Collected	WGS	Collected	WGS	Collected	WGS	Collected	WGS	Collected	
NSBL	47	327	37	277	39	208	0	8	3	11	1	7	127/838
ESBL	61	64	69	72	32	48	72	91	60	75	35	38	329/388
AmpC	9	9	4	4	1	1	13	15	6	6	15	15	48/50
AmpC-like	40	45	36	42	23	29	31	36	29	37	18	22	177/211
Susceptible	0	7	0	0	0	0	0	1	0	0	0	0	0/8
Total	157	452	146	395	95	286	116	151	98	129	69	82	681/1495

^a^Details on collected and sequenced isolates are provided in Table S1 and S8.

^b^ß-lactam resistance profiles (narrow spectrum β-lactamase (NSBL), extended spectrum β-lactamase (ESBL), AmpC, and AmpC-like, which corresponded mainly to *E. coli* chromosomal *ampC* derepression) were as previously described^35^ and reported in the methods section.

^c^Illumina whole genome sequenced.

**Fig. 2:**
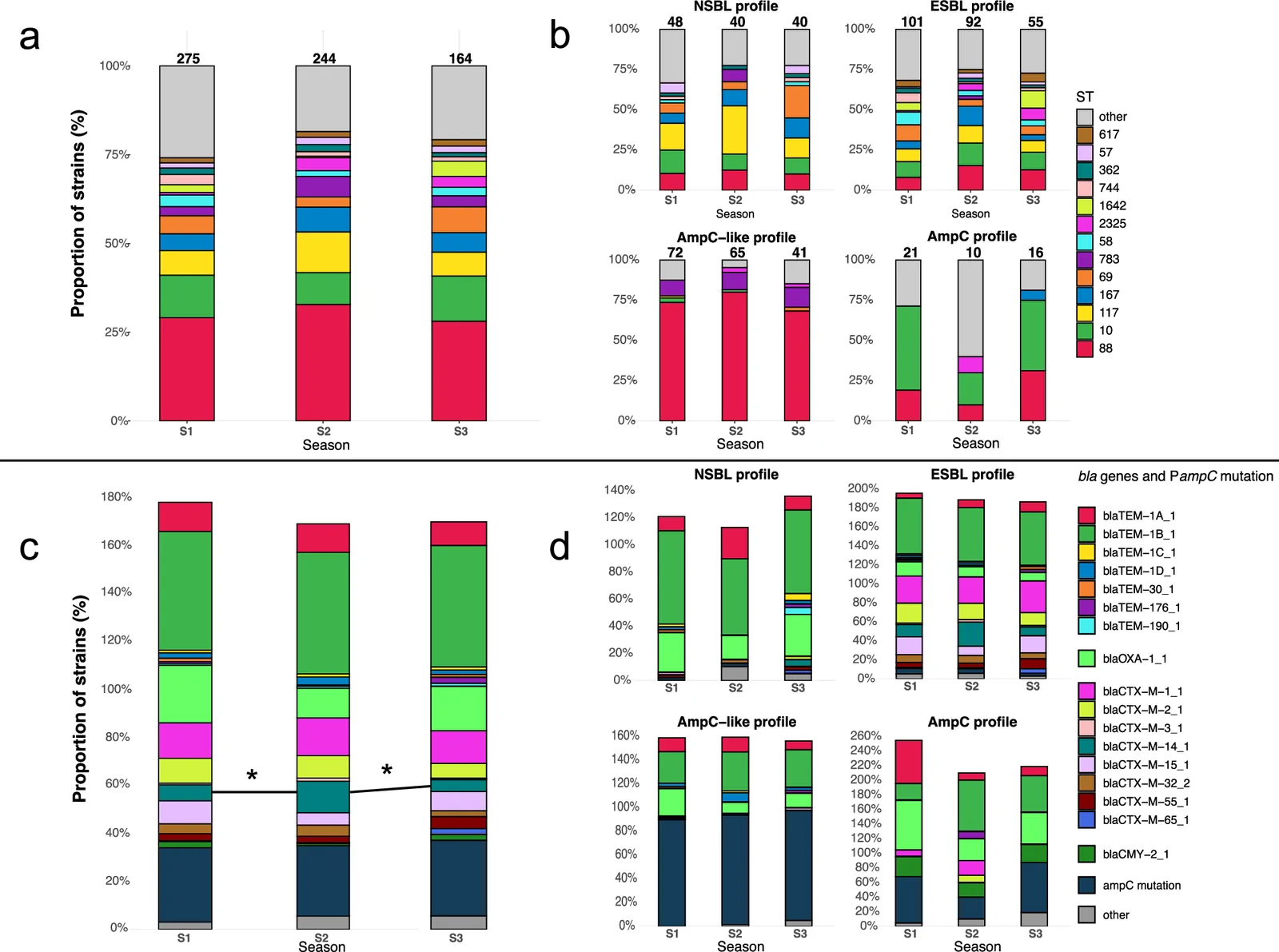
Sequence type and β-lactamase distribution according to season and β-lactam resistance profile. **a** Sequence type (ST) distribution of the 681 isolates according to the three calving seasons S1, S2, and S3. **b** ST distribution according to the four phenotype profiles: NSBL, ESBL, AmpC-like and AmpC. The number of isolates of each profile is indicated above the histogram. **c** β-lactamase gene and P_*ampC*_ mutation frequencies in the 681 isolates according to the three calving seasons. **d** β-lactamase gene and P_*ampC*_ mutation frequencies according to the four β-lactam resistance profiles: NSBL, ESBL, AmpC-like and AmpC. Percentages correspond to the percentage of isolates carrying the β-lactamase gene or the P_*ampC*_ mutation, explaining why total numbers are higher than 100%. Statistical significance between the β-lactamase gene and ST frequencies was assessed with a global Kruskal–Wallis test. *p. < 0.05. No trend was identified over the three calving seasons. ST (**a**, **b**) and β-lactamase genes and P_*ampC*_ mutation (**c**, **d**) are colored according to the figure key.

The 681 isolates from season S1-3 belong to 77 different STs, with 78% of the isolates belonging to the 13 most abundant (Figs. 1 and 2a, b). Phylogroup C was dominant with 32% of the isolates, including 205 ST88 isolates and 25 from its double locus variant, ST783. Phylogroup A contains 26% of the isolates, with 80% belonging to clonal complex 10 (76 ST10, 39 ST167, and 11 ST617). The two most common STs in phylogroup B1 were ST58 (*n* = 17, 22%) and ST1642 (*n* = 14, 18%). Thirty-four of the 70 phylogroup D isolates belong to ST69 (49%) and nine to its single variant ST106 (13%). All phylogroup G isolates belong to ST117 (*n* = 57). Despite the decreased 3GC resistance rate previously reported^11^, the ST distribution of the isolates according to the four β-lactam resistance profiles did not change during the three seasons (Fig. 2a, b).

### 99% of *E. coli* isolates from S1 to S3 are MDR and exhibit a stable β-lactamase gene content

The number of acquired β-lactamase genes among isolates from seasons S1-3 ranged from none in 79 isolates to four in two isolates (Table S1). 206 isolates (30%) had a mutation in the chromosomal P_*ampC*_ promoter at position −42 or −32 or a TA insertion between positions −13 and −14. These mutations were shown to derepress *ampC* expression^17,18^.

The *bla* gene content combined with P_*ampC*_ mutations was largely consistent with the β-lactam resistance profile (Fig. 2c, d and Table S1). Among the 127 NSBL-producing isolates, *bla*_TEM-1_ variants and *bla*_OXA-1_ were predominant, present in 88% and 28% of the isolates, respectively. All except two of the 329 isolates showing an ESBL phenotypic profile carried a *bla*_CTX-M_ gene (Fig. 2d and Table S1). Six isolates carrying a *bla*_CTX-M_ gene showed an AmpC (*n* = 4) or NSBL (*n* = 2) profile. *bla*_CTX-M-1_ was the most frequent gene (30%), followed by *bla*_CTX-M-2_ (19%), and *bla*_CTX-M-14_ and *bla*_CTX-M-15_ (17% each). The AmpC-like phenotype (*n* = 177) was mainly caused by mutations in the promoter region of P_*ampC*_, which were found in 90% of the isolates (*n* = 170). Four AmpC-like isolates harbored an acquired AmpC-encoding gene, *bla*_CMY-2_ (*n* = 1) or *bla*_DHA-1_ (*n* = 3). Among the 48 isolates with an AmpC profile, 16 harbored an AmpC-encoding gene: *bla*_CMY-2_ (*n* = 13), *bla*_DHA-1_ (*n* = 2) or both (*n* = 1); 28 isolates had mutations in P_*ampC*_ with 26 harboring acquired β-lactamase genes, predominantly *bla*_OXA-1_ (*n* = 18); four carried *bla*_CTX-M-1_ combined with one or two other β-lactamase genes (Table S1).

In addition to β-lactamase genes, isolates carried a wide variety of ARGs, with a median of 10, ranging from none in one isolate up to 21 in two isolates. We identified mutations in the quinolone resistance-determining regions (QRDR) of *gyrA* and/or *parC* in 67.4% of the isolates (*n* = 459). The median number of ARGs ranged from eight for AmpC-like isolates to 14 for those with an AmpC phenotype (Fig. S2). There was no carbapenemase gene, in agreement with the absence of carbapenem-resistant isolates. Fifty-four isolates from 18 different STs carried an *mcr* gene. The vast majority expressed *mcr-1* (*n* = 50), two expressed *mcr-2* and two *mcr-9*. As previously reported^19^, *mcr-9* conferred a lower colistin MIC compared to *mcr-1* and *mcr-2* (Table S3). *mcr*-carrying isolates carried a larger number of ARGs (median = 13) than non-*mcr*-carrying isolates (median = 10) (*p* < 0.0001).

Despite the modification of antibiotic usage, no statistically significant trend was observed during the three calving seasons studied for the mean ARG number, β-lactamase gene diversity and number of P_*ampC*_ mutations when considering the four different β-lactam resistance profiles (Fig. 2c, d). These results are in agreement with a preliminary study based on a triplex PCR reaction targeting the three main *bla*_CTX-M_ groups: 1, 2, and 9^14^.

### Mutations in P_*ampC*_ were predominant among phylogroup C isolates

**Fig. 3:**
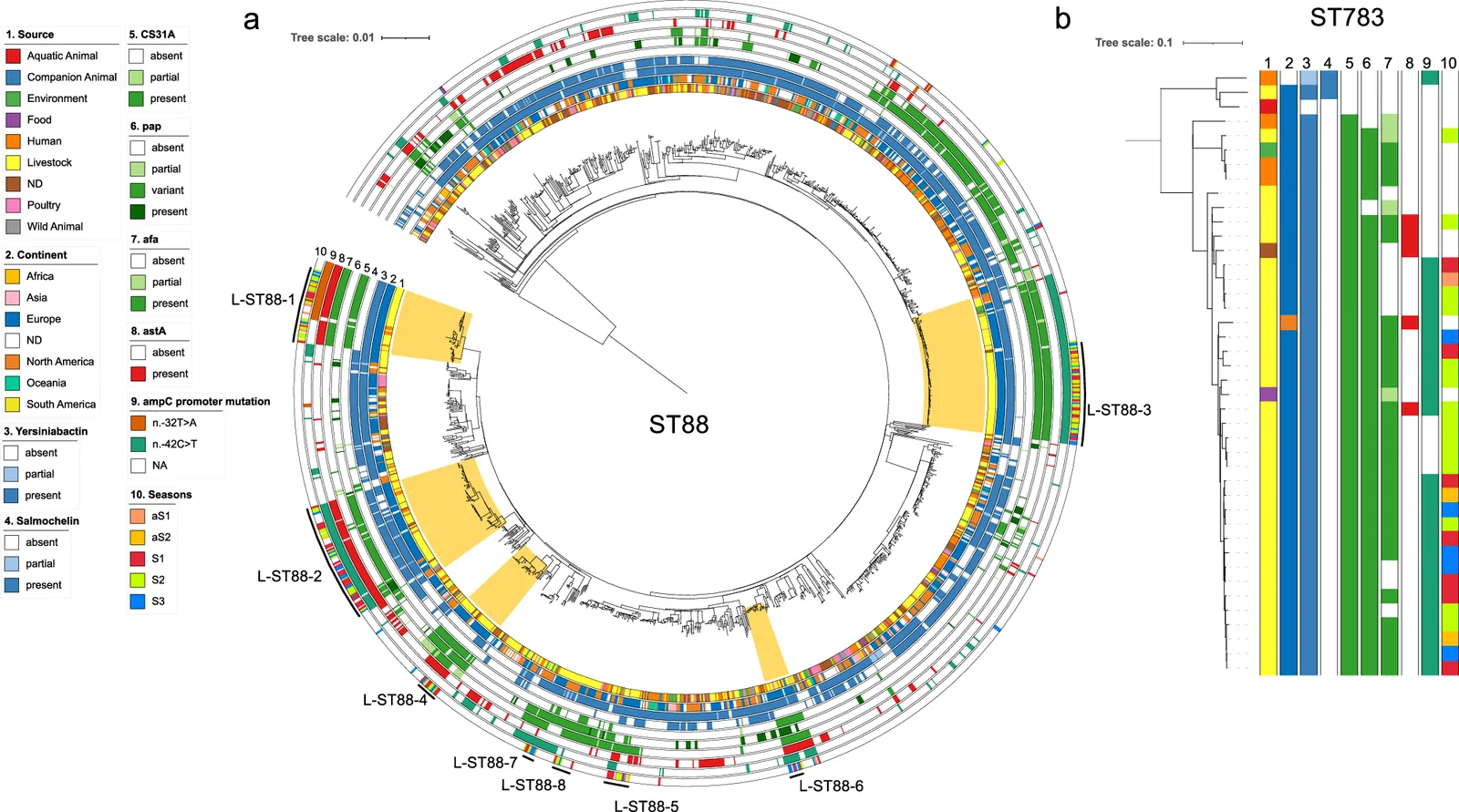
Phylogeny and characteristics of ST88 and ST783 *E. coli* isolates from the five seasons combined with EnteroBase isolates. **a** ST88 isolate from the study (*n* = 222) combined with 1283 EnteroBase isolates. **b** ST783 isolates from the study (*n* = 29) combined with 13 EnteroBase isolates. Maximum likelihood phylogeny was performed by using RAxML 8.2.12^45^ based on recombination-free core genome alignment, computed with Snippy 4.6.0 (https://github.com/tseemann/snippy) and Gubbins 2.4.1^46^ to filter recombined regions. Genomes were annotated according to the figure key as circles from inside to outside (ST88) or from left to right (ST783): the source niche, the continent of origin, yersiniabactin, salmochelin, CS31A, *pap*, *afa*, *astA*, P_*ampC*_ mutation and the season and visualized with iTOL v7^47^.Lineages emerging following the acquisition of virulence factors are colored in yellow. Clusters reported in Table 2 are indicated outside the last circle.

Among the 206 isolates of seasons S1-3 showing a mutation in P_*ampC*_, 177 (86%) belonged to phylogroup C (157 to ST88 and 20 to ST783). Similarly, P_*ampC*_-mutated isolates represented 76% and 77% of the isolates from these two STs, respectively. During the two previous calving seasons aS1 and aS2, 17 out of 83 isolates (20%) showed mutation in P_*ampC*_. All belonged to phylogroup C (12 of the 19 ST88 isolates, three of the four ST783 isolates and two of the three ST23 isolates). To contextualize the acquisition of these mutations, we performed whole-genome phylogeny by including 1243 ST88 and 14 ST783 genome sequences retrieved from EnteroBase (Fig. 3). Among them, 222 ST88 (18%) and 4 ST783 (29%) isolates showed a mutation in P_*ampC*_. Among the ST88 isolates from EnteroBase, P_*ampC*_ mutations were more frequently associated with livestock (31% of the 426 isolates) than with humans (12% of the 268 isolates) (*p* < 0.001). The 24 isolates from the study, mutated at position −32 (T>A) in P_*ampC*_, were monophyletic (bootstrap value of 100) and belonged to ST88. This lineage (L-ST88-1) includes 14 isolates from EnteroBase (Fig. 3 and Table 2). Two large lineages, likely monophyletic, L-ST88-2 (74 isolates, bootstrap value of 100) and L-ST88-3 (63 isolates, bootstrap value of 89), share the same mutation at −42 (C > T) in P_*ampC*_. 24 additional ST88 isolates clustered in seven lineages of one to seven isolates showed this mutation. Among the 42 ST783 isolates, 26, including 23 from our study, share the −42 C > T mutation in P_*ampC*_. Based on the tree structure (Fig. S3b), several independent mutation events in P_*ampC*_ might have occurred.

**Table 2:** Monophyletic lineages gathering ^a^deduplicated isolates from the study

ST	Lineage	Bootstrap value %	^b^Isolates	^b^Study isolates	^c^Origin of EnteroBase isolates	^b^Human isolates
ST10	L-ST10-1	87,2	12	10	UK (1, farm) Italy (1, Bovine)	-
	L-ST10-2	99,7	15	15	-	-
	L-ST10-3	100	17	17	-	-
	L-ST10-4	100	10	10	-	-
ST57	L-ST57-1	100	6	5	Germany (1, bovine)	-
	L-ST57-2	100	8	5	Germany (1, animal; 1, human), France (1, Human)	2
ST88	L-ST88-1	100	52	36	the Netherlands (7, bovine; 5 human), Spain (2, human), Belgium (1 human), Germany (1 human)	9
	L-ST88-2	100	69	47	USA (3, bovine), Ireland (1, human; 1, bovine), France (1, human), Belgium (1, Human; 2, swine; 4, bovine), Switzerland (1, human), Spain (2, human), the Netherlands (3, human), Unknown (2)	9
	L-ST88-3	89	63	63	-	-
	L-ST88-4	100	14	10	Germany (2, human), Luxembourg (1, bovine), the Netherlands (1, human)	3
	L-ST88-5	98	13	11	France (1, human), Luxembourg (1, food)	1
	L-ST88-6	100	14	7	France (2, bovine; 1, human; 1, poultry), Switzerland (2, human), UK (1, human)	4
	L-ST88-7	100	5	5	-	-
	L-ST88-8	98	5	5	-	-
ST106	L-ST106-1	75	7	7	-	
ST117	L-ST117-1	98	50	44	France (1, human;1, bovine), Netherlands (1, human), Luxembourg (1, swine), Sweden (1, human), USA (1, bovine)	3
	L-ST117-2	100	15	14	UK (1, Human)	1
	L-ST117-3	100	6	5	North America (1, human)	1
ST167	L-ST167-1	96	15	14	1 (unknown)	-
	L-ST167-2	100	8	8	-	-
	L-ST167-3	90	12	8	Germany (2, human), the Netherlands (1, human), the UK (1, Human)	4
ST345	L-ST345-1	99,6	6	6	-	-
ST457	L-ST457-1	98,6	8	5	Luxembourg (3, Livestock)	-
ST617	L-ST617-1	99,8	14	10	Germany (3, Human) Luxembourg (1)	3
ST783	L-ST783-1	99,4	32	27	France (2, bovine), Poland (1, bovine), Canada (1, bovine), Luxembourg (1, food)	-
ST1642	L-ST1642-1	99,9	13	13	-	-
ST2325	L-ST2325-1	100	8	8	-	-
	L-ST2325-2	100	5	5	-	-
Number of isolates			502	420	82	40

^a^Only one isolate was considered for clonal isolates from the same animal.

^b^Number of isolates.

^c^Country or origin, in parentheses, the number of isolates and the source.

Among other phylogroups, we detected two additional monophyletic lineages of 18 ST10 (Fig. S3a) and six ST345 (Phylogroup B1) (Fig. 1) isolates.

We analyzed polymorphisms in regions 10 kb upstream and downstream of *ampC* to determine if the P_*ampC*_ mutation was acquired by recombination from a mutated strain. The cluster of 18 ST10 isolates mutated in P_*ampC*_, showed only two SNPs in this 21 kb region compared to the L-ST88-2 isolate R0092. The acquisition of the mutation in P_*ampC*_ resulted from a recombination event from a mutated ST88 strain. In other cases, we did not obtain evidence of P_*ampC*_ mutation acquisition by recombination (Fig. S4).

### A majority of *E. coli* isolates cluster in monophyletic lineages frequently also encompassing human isolates

To further characterize relationships between *E. coli* isolates from different herds, we analyzed phylogenies of the 13 STs with more than ten isolates, contextualized by including EnteroBase genomes (Fig. 3; Figs. S3 and 5–7). Visual inspection of phylogenetic trees showed that many isolates from calves formed distinct lineages with few EnteroBase isolates. Using as criteria, lineages with at least five isolates from the study, fewer than half of the isolates from EnteroBase and a bootstrap value higher than 75%, we identified 28 monophyletic lineages from 12 STs. These lineages comprised 420 non-duplicated isolates from the study (54%) and 82 isolates from EnteroBase (Table 2). Twelve lineages included only isolates (*n* = 5 to 63) from the study. Only six EnteroBase isolates (7%) came from outside Europe (North America). Eight isolates were from Belgium, and most other isolates were from countries bordering Belgium (Table 2). Thus, these lineages showed a limited geographic diffusion. 49% of EnteroBase isolates were of human origin (*n* = 40), suggesting host jumps from bovine to human host. This was supported by ancestral state reconstruction by using PastML^20^. It predicted cattle as the most likely host of the ancestral strain of ten of the 11 lineages containing human isolates and 18 bovine host to human host transitions with a probability higher than 0.8 (Fig. S8). All EnteroBase isolates, as those from this study, were predicted as MDR and carried a median of nine ARG per isolate, 51% had a P_*ampC*_ mutation, 83% had *gyrA*/*parC* QRDR mutations and two human isolates carried the carbapenemase gene *bla*_OXA-48_ (Table S4).

### Short-term dissemination of *E. coli* occurred mainly at short geographic distances

**Fig. 4:**
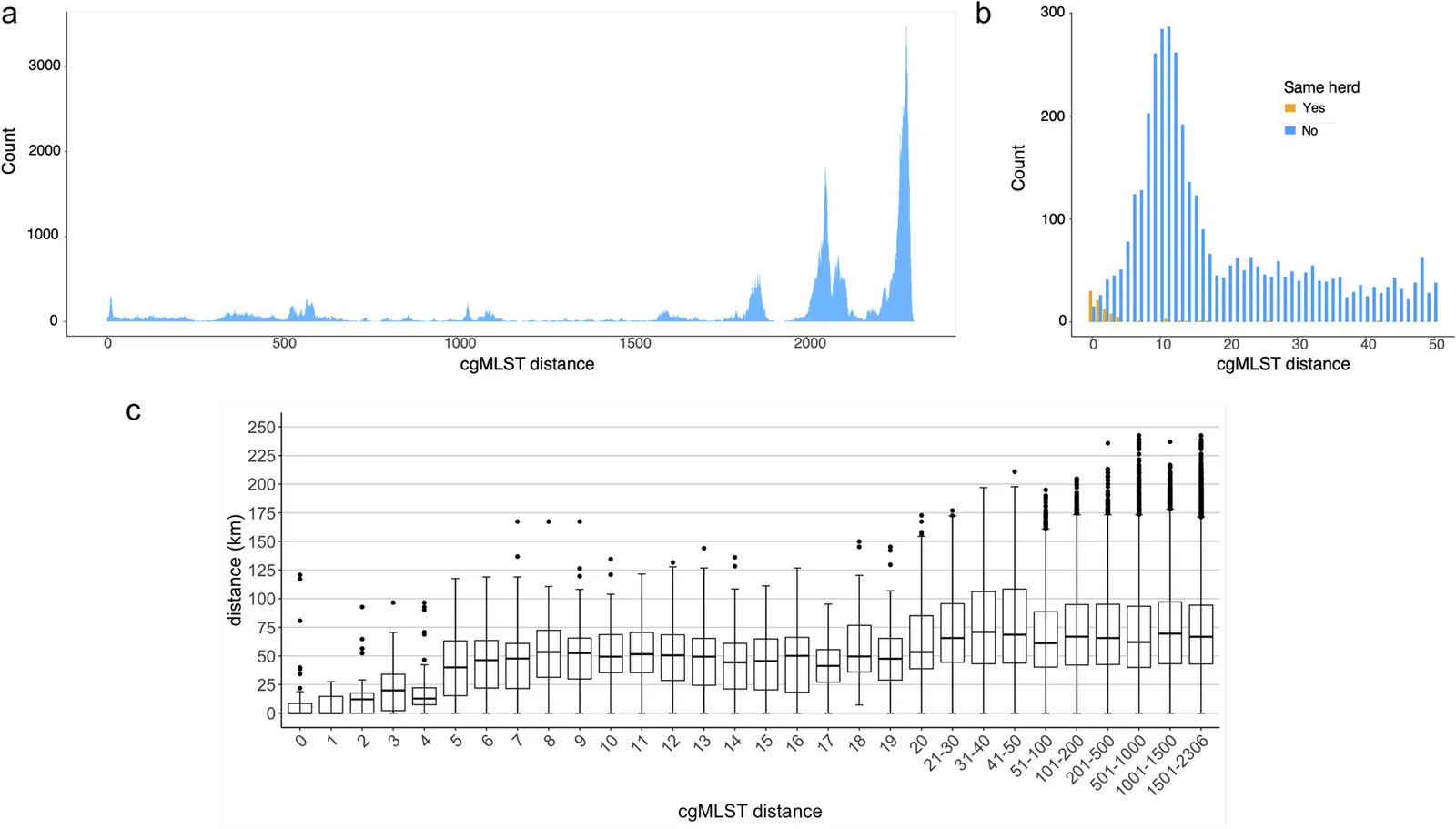
Genetic and geographic distances between isolates. **a** distribution of the pairwise genetic distances in the number of different cgMLST alleles for all pairs of deduplicated isolates from different herds. **b** Same as (**a**), but for pairs with 0 to 50 different alleles. In orange, pairs of isolates from the same herd. **c** Relation between the genetic distance between two isolates in number of different alleles and the geographic distance in km.

In complement to maximum likelihood (ML) phylogeny, we computed the cgMLST distances of the 291,466 pairs of isolates, after deduplicating identical isolates from the same animal (Fig. 4). It revealed a peak centered at a cgMLST distance of ten different alleles. 511 isolates had a cgMLST distance of less than 21 alleles with at least one other isolate (Fig. 4b and Table S5). Among these isolates, 354 belonged to the 28 lineages identified on the phylogenetic trees, representing 84% of the isolates from these lineages. A similar analysis restricted to pairs of isolates from the same herd showed a similar peak of closely related isolates with 98 isolates with less than five different alleles (Fig. 4b and Table S5). To estimate the rate of dissemination, we computed the genetic distance between isolates as the number of different alleles by cgMLST and the geographic distance between farms (Fig. 4c). We observed two jumps in the distance distribution. Isolates from the 254 pairs differing by less than five alleles were at a median distance of 10 km, and 203 (80%) were from herds less than 25 km apart. This suggested a local spread of resistant *E. coli* between these farms. Isolates from pairs differing by 5 to 20 alleles were at a median distance of 49 km compared to 66 km for isolates differing by over 20 alleles. This observation suggests other longer-term constraints on *E. coli* dissemination.

**Fig. 5:**
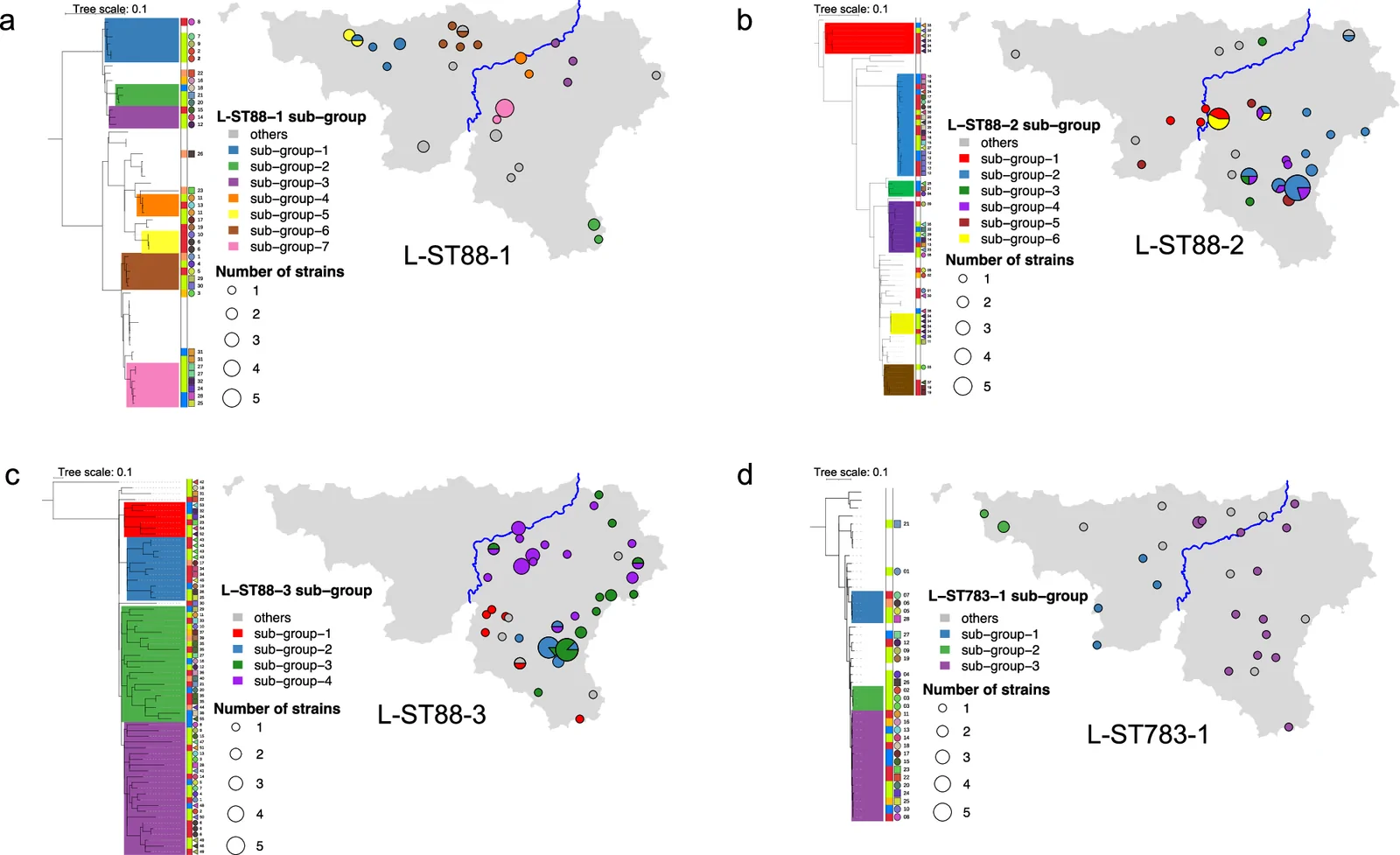
Local dissemination of closely related isolates and geographic specificity. The geographic localization was compared to the phylogenetic proximity by localizing isolates according to the GPS coordinates of the postal code. The size of the disk corresponds to the number of isolates. Colors correspond to tree sublineages and as sectors if more than one sub-lineage. Isolates were annotated on the right of the tree according to the figure key. From left to right, the season (in white isolates from EnteroBase) and the herd. The blue line represents the Meuse. **a** L-ST88-1, isolates were from the whole of Wallonia, with local clustering of seven sub-lineages. **b** Lineages L-ST88-2, 34 isolates were from herds east of the Meuse, and 7 were from herds west. **c** Lineage L-ST88-3 all isolates were from herds east of the Meuse. **d** Lineage L-ST783-1 isolates were from the whole of Wallonia, with local clustering of three sub-lineages.

To further analyze isolate dissemination, we localized on the map of Wallonia those belonging to 11 lineages with more than nine isolates from our collection. Isolates closely related in some lineages are clustered geographically. This is the case for sub-clusters of isolates in L-ST-88-1 (*n* = 6), L-ST88-2 (*n* = 7) and L-ST88-3 (*n* = 3) (Fig. 5), and in four other STs: 10, 167, 617, 1642 (Figs. S9and S10). Our definition of lineage was independent of the evolutionary branch length, explaining why local dissemination was variable among these lineages. This analysis revealed, in addition to the geographic proximity of isolates with low genetic distance, differences between east and west of the River Meuse. All isolates from lineages L-ST88-3 and most of L-ST88-3 were from herds located in the eastern part of Wallonia. Two ST783 sublineages were restricted to the west of the Meuse, whereas the third one was mainly in the east (Fig. 5d). This geographic distribution might correspond to the jump in distances observed for pairs with over four different alleles (Fig. 4c).

### The population of *E. coli* infecting calves is different east and west of the Meuse

**Fig. 6:**
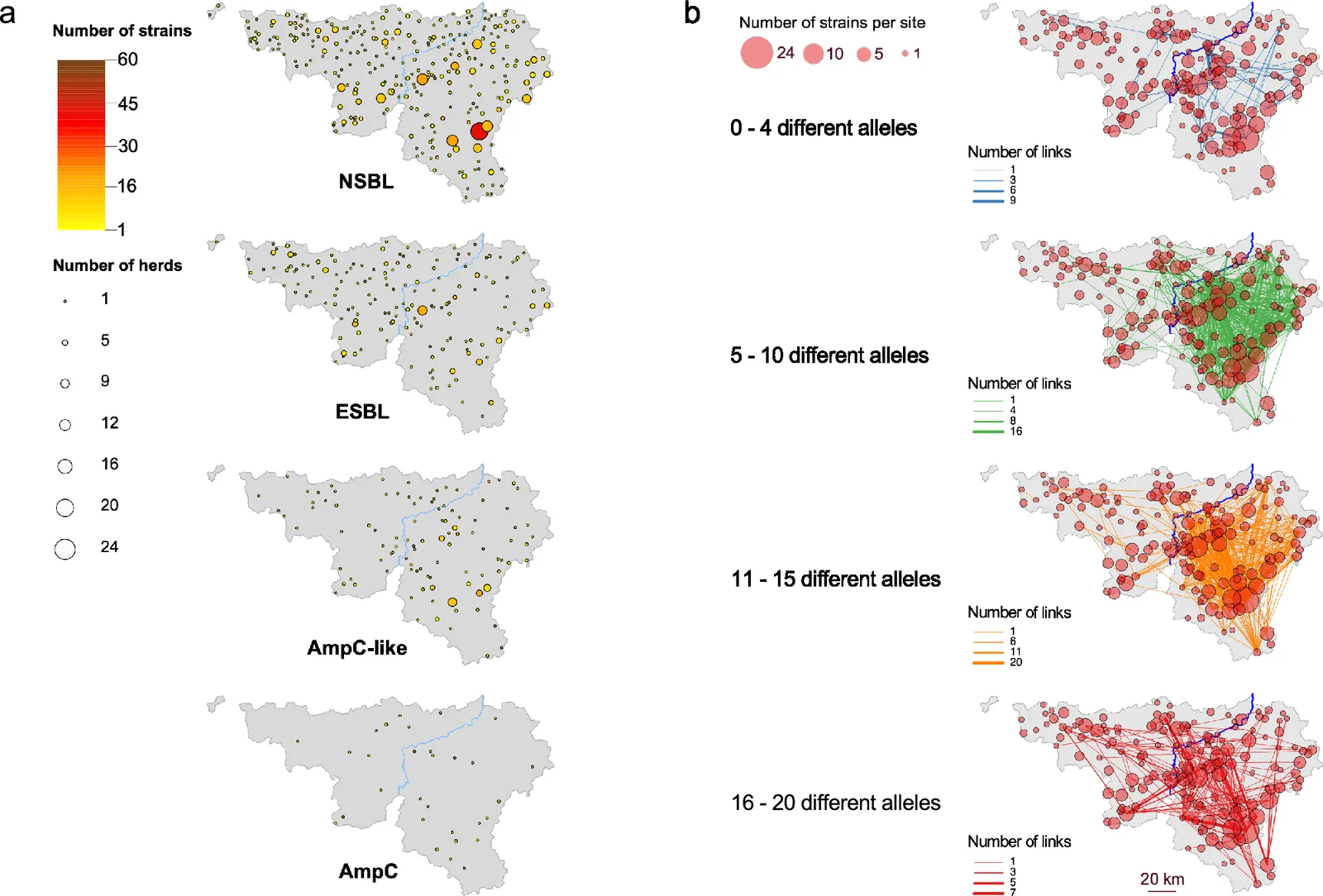
β-lactam resistance profiles and differences in transmission between west and east of the Meuse. **a** Geographic distribution of the isolates belonging to the four β-lactam resistance profiles. In each postal code, the number of isolates and the number of herds were indicated by the color and the diameter of the disk, respectively, according to the figure key. **b** Geographical links of isolate pairs showing less than five different alleles by cgMLST (blue links), between five and ten different alleles (green links), between 11 and 15 (orange links) and between 16 and 20 (red links). Circle diameters represent the number of isolates per postal code and the thickness of the lines, the number of isolate pairs according to the figure key.

To further quantify the impact of the geography on transmission, we analyzed the geographic distribution of isolates from seasons S1-3 (Fig. 6a). Among the 1495 isolates we have phenotypes (Table S1), 813 isolates came from 445 herds located east of the Meuse, 654 isolates from 370 herds west of the Meuse (Tables S6). 26 isolates were from 14 farms located in postal codes crossed by the Meuse. The distribution of β-lactam resistance profiles was significantly different between east and west of the Meuse. The frequency of AmpC-like isolates was twice as high in the east as in the west, while the ESBL isolates were more frequent in the west. The proportion of NSBL and AmpC profiles was similar between east and west (Table S6). These differences were also observed for sequenced isolates from the five calving seasons. In agreement with the dominance of AmpC-like profiles in the east, we observed a proportion of ST88 isolates more than double compared to the west. However, the proportion of ST88 isolates not part of a cluster was similar between east and west, with 2.7% and 2.8% of the sequenced isolates, respectively (Table S6).

Next, we analyzed the geographic distribution of isolate pairs between farms from the five calving seasons, showing less than 21 different alleles by cgMLST (Fig. 6b and Table S5). We considered all isolates, only ST88 isolates, only isolates with an AmpC-like profile, and conversely all isolates except ST88 or except AmpC-like profiles and performed the statistical analysis on the number of isolates belonging to at least one pair (Table S7). After normalization by the number of isolates, we observed ten times more pairs with fewer than 21 different alleles on the east compared to the west of the Meuse. This was largely attributable to isolates with an AmpC-like profile or from ST88. However, the difference between east and west was still significant when excluding AmpC-like or ST88 isolates. When considering transmissions within a farm, there was no difference between east and west. These findings confirmed the observation made on individual clusters. Local geography (east or west of the Meuse) had a strong impact on the phenotype of the collected isolates and on the spread of *E. coli*.

### The acquisition of virulence factors shaped the *E. coli* population in sick calves

*E. coli* isolates came from diarrheic animals or those showing invasive infections. They were potentially pathogenic. We analyzed their genomes for the presence of virulence-associated genes, focusing on three different classes: adhesins/pili, toxins and siderophores (Table S8 and Fig. S11). For comparisons, we performed a similar analysis on EnteroBase isolates of the dominant STs (Table S9). A P-fimbriae-like operon was the most frequent (*n* = 344 isolates, 45%). In this operon, *papG* is replaced by a protein annotated as “PapG chaperone-binding domain”, which might similarly act as an adhesin. This locus was rarer among EnteroBase isolates, even among livestock isolates (Table S9). These observations suggest a role in the virulence in calves, as shown for P-fimbriae carrying the PapG adhesin in human infections^21^. The *afa* locus encoding the Diffuse Adherence Fibrillar Adhesin associated with Enteropathogenic *E. coli*^22^ was present in 324 isolates (42%) and was similarly much more abundant than among EnteroBase isolates. The *clp* locus (complete or partial) encoding CS31A was present in 272 isolates and was more frequent than among EnteroBase isolates. A *f17* locus, complete or partial, was present in 114 isolates. The *fanCDEGH* operon encoding F5 (K99) pili was present in 18 isolates (15 ST10 and 3 ST120). The agreement between the genomic detection of the genetic loci and the agglutination assay following growth on the MINCA plate was variable, from high in the case of F5 to low in the case of F17, as further presented and discussed in Supplementary Results. The siderophore aerobactin locus and the *sit* operon (iron import) were more frequent than among EnteroBase isolates, whereas salmochelin was less abundant. Shigatoxin (*n* = 5), α-hemolysin (*n* = 12) and *cnf* (*n* = 6) loci were rare in our collection. Only the heat-labile toxin AstA^23^ (*n* = 215, 28%) was prevalent and more frequent than in EnteroBase isolates (Table S9). Based on the detection of virulence factors, 163 isolates were defined as enterotoxigenic (ETEC), six as enterohemorrhagic (EHEC), five as enteropathogenic (EPEC) and one as shigatoxigenic (STEC) (Table S8). These virulence factors were more frequent in phylogroups C, D and G compared to other phylogroups.

We next focused our analysis on four dominant STs: ST10, ST117 and ST88/ST783 (Fig. 3 and Fig. S3). A visual inspection of the trees revealed, except in ST10, an association of some virulence factors and lineages we previously identified. For example, isolates from lineage L-ST-88-1 share the adhesins CS31A, the Pap-variant and the thermostable Enterotoxin EAST1. The acquisition of the P_*ampC*_ mutation occurred later in a sublineage of L-ST88-1 (Fig. 3). Similarly, ST783 isolates were enriched in the CS31A, Pap-like and Afa adhesins loci. All except two ST117 isolates carried the CS31A (*clp*) locus. On the other hand, the salmochelin operon was counter-selected among the calf isolates (Table S9). This operon was absent from all except two ST117 isolates from the study, but frequent among EnteroBase ST117 isolates (Fig. S3). Therefore, virulence contributed to shaping the population of *E. coli* in sick calves.

## Discussion

*E. coli* is a major cause of deadly infections in calves, resulting in economic losses for farmers. Thanks to our extensive sampling of *E. coli* throughout Wallonia, we were able to answer key questions regarding the spatiotemporal dynamics of the *E. coli* population infecting calves at a country level. The restriction of antibiotics essential for human health in Belgium^10^ led to a reduction in 3GC-resistant isolates (here, the ESBL and AmpC profiles) and an increase in isolates still resistant to amoxicillin but not to 3GC^11^. Thanks to our selection for WGS of a balanced but non-proportional collection, we showed that, considering the four β-lactam resistance profiles during the three calving seasons from 2017 to 2020, there was no significant change in the population structure based on ST, nor a significant impact on β-lactamase gene frequencies (Fig. 2). This stability suggests that antibiotics select for resistance in a bacterial population adapted to a host and do not shape the population.

The specificities of the isolates we have analyzed were that they originated from sick calves and were ampicillin-resistant. The origin from sick animals was associated with an enrichment in virulence factors compared to isolates from EnteroBase (Table S9). This enrichment was prevalent among isolates from phylogroups C, D and G. Although the isolates were selected only as resistant to amoxicillin, which is common among *E. coli*, all except nine isolates were predicted to be MDR, and only 25 isolates were resistant to less than four antibiotic classes. Surveillance in Belgium of *E. coli* based on carriage of beef under one year of age indicates a rate of 50% of MDR isolates^24^. Considering that our isolates belonged to the 85% of amoxicillin-resistant isolates, the 99% rate we observed in sick animals was still considerably higher. This could be explained by the number of antibiotic treatments administered to calves^5^, which may have led to a link between virulence and resistance.

ST88 represented almost 30% of all sequenced isolates in our study. ST88 isolates have been frequently isolated from bovine hosts and were previously reported as mutated in P_*ampC*_ both in animals and humans^25,26^. We similarly observed a strong association between derepression of *ampC* and ST88, with 75% of the 223 isolates mutated in P_*ampC*_ belonging to this ST. This association could result from a higher level of resistance due to AmpC overproduction (better selection) or from a lower fitness cost (better dissemination) in ST88 strains. Furthermore, ST88 isolates of the study carried more virulence genes than EnteroBase isolates (adhesins, siderophores and the AstA toxin). In contrast, the salmochelin locus was counterselected. ST88 isolates from the study and of the closely related ST783 clustered mainly in a few lineages associated with these genetic traits (Fig. 3). The P_*ampC*_ mutations were subsequently acquired by sublineages within these lineages, confirming that the major driving force for the selection of these lineages was likely virulence.

ST10 was the second most abundant ST with 81 isolates. Surprisingly, 89% of the ST10 isolates from the study clustered with a cgMLST distance lower than 100, together with only 2.6% of ST10 isolates from EnteroBase. EnteroBase isolates in this lineage were mainly from animals (182 versus 63 from humans) (Fig. S3a). Compared to the bulk of ST10 strains, this cluster might be adapted to the bovine host and prone to being pathogenic. However, we did not detect a strong enrichment for the virulence factors we have analyzed (Fig. S3a). Further studies will be needed to identify a specific adaptation of these ST10 strains to young calves, if any.

The high frequency of ST117 (72 isolates) was unexpected, since ST117 is an avian pathogenic lineage associated with poultry^27,28^. 89% of the ST117 isolates from our study belong to three lineages together with seven EnteroBase isolates: four from humans, one from bovine and one from swine, but none from birds. These three lineages have acquired the *clp* operon (CS31A) and are enriched in the *pap*-like operon. They have also lost the salmochelin operon (Fig. S3b). Therefore, these pathogenic lineages likely adapted to the bovine host have been subsequently transmitted to other hosts.

Across our collection, 54% of the isolates, when combined with EnteroBase isolates, are grouped into lineages (Table 2). Half of the EnteroBase isolates from these clusters were of human origin. As we predicted, mainly transitions from bovine to human hosts (Fig. S8), these highly resistant lineages, bovine-adapted, might represent a long-term reservoir for human MDR *E. coli* strains.

These lineages are associated with a limited geographical dissemination. 93% of the EnteroBase isolates from these clusters were from European countries, compared with 47% (3442 out of 7282) for all EnteroBase isolates belonging to the 13 most abundant STs in the study. When we focused on short evolutionary distances (less than five different cgMLST alleles), we observed that isolates were from farms at a median distance of 10 km (Fig. 4). Animal transfers between farms in Wallonia are likely to be rare, as 94% of the calves sent to rearing farms were sent to farms in Flanders^29^. Indirect local transmission via vehicles traveling between farms or direct contact between animals in the fields of neighboring farms are likely routes of transmission. Dissemination of Shiga toxin-producing *E. coli* O157:H7 among dairy farms in close geographic proximity was similarly described in Northeast Ohio, USA^30^.

We observed very different behavior between the two areas in Wallonia separated by the Meuse. Clusters of geographically and genetically closely related isolates were abundant in the east. While transmissions were less frequent in the west and between east and west (Fig. 6). This difference was mainly due to the geographic distribution of lineages of isolates with an AmpC-like profile dominant in the east (Table S6). Conversely, ESBL isolates were more abundant in the west. Different factors might contribute to the observed differences between the two parts of Wallonia, which might include different antibiotic usage and farming practices^31,32^.

We acknowledge some limitations in our study. Only β-lactam-resistant isolates were analyzed. However, the rate of ampicillin-susceptible isolates was low, ranging from 11.1 to 16%^11^ during the study period. Therefore, our analysis remains largely representative of the circulating strains and provides an accurate view of resistant isolates. Here, we have analyzed isolates from sick animals. A similar analysis of carriage isolates would allow us to better define the specificities of the *E. coli* population in sick calves and to evaluate the circulation of pathogenic isolates among healthy animals. We also did not analyze *E. coli* isolated from the environment to confirm its contribution to the between-farms dissemination of MDR *E. coli*. Our study relied on short-read sequencing, which prevents us from analyzing the contribution of plasmid transmission to the virulence and resistance dynamics. Our systematic sampling started in November 2017, and the restriction on 3GC started in 2016. However, reduction of 3GC resistance was also observed during the three seasons we have analyzed^11^. Antibiotic usage per herd or per postal code would allow us to determine its contribution to the difference between west and east of the Meuse. However, these data are not available to our knowledge.

Our analysis showed that a combination of virulence in calf, antibiotic resistance and local dissemination all possibly contribute to the observed resilience of calf-infecting *E. coli* in terms of population and β-lactam resistance mechanisms. It also suggests host jumps from bovines to humans within the locally disseminated MDR lineages. The extensive geographic sources of the isolates demonstrate the importance of local transmission of *E. coli* and reveal striking differences between the east and west of Wallonia. It provides means to reduce transmission by limiting the transfer of contaminated material between closely related farms, as done prior to limiting the transmission of the viral foot and mouth disease^33^.

## Methods

### *E. coli* isolates, growth conditions, and antibiotic susceptibility testing

*E. coli* isolates were obtained from young calves of Walloon farms. They were isolated on Gassner agar plates (Thermo Fisher Scientific), either from fecal samples of diarrheic calves in farms or from intestinal contents or internal organs of calves suffering from diarrhea or septicemia and necropsied the ARSIA laboratory as previously described^14^. Isolates from internal organs were considered pathogenic. Three *E. coli* colonies from feces or intestinal contents were streaked on (i) MINCA agar plates to test the production of *fimbriae* F5 and/or F17 by agglutination tests (Biovac, Beaucouzé, France) and for the CS31A surface antigen, (ii) Enterohemolysin (EHLY) agar plates containing washed sheep blood erythrocytes (Oxoid) to test the production of an enterohemolysin (Ehly), and (iii) only for seasons S1-3 (2017-2020) on MacConkey-CTX - lactose plates complemented with 1 mg/L cefotaxime (Led Techno). Isolates positive for the surface antigen(s) and for enterohemolysin were considered pathogenic. Other isolates were considered non-pathogenic, but only those growing on MacConkey-CTX were kept. Temporal analysis was performed considering only isolates from seasons S1-3. The 83 isolates from seasons aS1 and aS2 sequenced to extend the diversity of our collection were isolated from organs or positive for at least one of the virulence factors tested. Antibiotic susceptibility testing for resistance against eight β-lactams (Amoxicillin, AMX; Amoxicillin/Clavulanate, AMC; Ceftiofur XNL; Cefquinome, CFQ; Cefotaxime, CTX; Cefotaxime/Clavulanate, CTC; Cefoxitin, FOX; Meropenem, MER) was performed by disk (I2A, France) diffusion assay following the EUCAST/CASFM-vet guidelines^34^. Isolates were classified in four resistance profiles according to patterns presented in Table S10. Colistin susceptibility was determined by microdilution using Sentititre plates (Thermo Fisher Scientific) following the manufacturer’s instructions.

We selected 681 *E. coli* isolates for WGS from 1495 non-redundant isolates from seasons S1-3^11,14^. They were isolated from the feces of diarrheic calves (*n* = 962) and from internal organs (*n* = 272) or the intestinal content (*n* = 261) of calves necropsied at ARSIA (Table S1). Isolates from internal organs or with positive agglutination test for fimbriae F5 or F17, the CS31A antigen (Biovac, France), or enterohemolysin-producers on washed sheep blood agar plates were considered pathogenic^14^. The 361 remaining isolates from feces or intestinal content were positive only on McConkey agar plates complemented with 1 mg/L cefotaxime and considered here as non-pathogenic (Table S1)^14^. Isolates were classified into four β-lactam resistance profiles: NSBL, amoxicillin resistant only; ESBL, 3GC resistant with clavulanic acid synergy; AmpC, 3GC and 4GC resistant without clavulanic acid synergy; and AmpC-like, amoxicillin resistant with no or reduced clavulanic synergy and cefoxitin resistant^11,35^ (Table 1 and Tables S1).

Sequenced isolates were selected for WGS to keep the balance between the four β-lactam resistance profiles (NSBL, ESBL, AmpC and AmpC-like) across the three calving seasons (Table 1). They were from 379 farms spread out over Wallonia (Tables S1). There were up to 13 farms per postal code and 1 to 11 sequenced isolates per farm (Fig. S1). Two isolates from different anatomic origins or from fecal material but showing different phenotypic profiles were selected from 68 calves, and three isolates from one calf. To obtain more temporal data, we selected 83 additional isolates, considered pathogenic according to our criteria, from calving seasons 2014-2015 and 2015-2016 (aS1, aS2) (Table S2). Fifteen were from herds already sampled during seasons S1-3, and 68 from 65 additional farms, making a total of 444 herds.

To comply with data privacy laws, animal and herd identifications were encoded at the data source before analysis.

### Whole genome sequencing, sequence assembly, and analyses

DNA extractions were performed with the Qiagen DNeasy Blood & Tissue Kit. Sequencing libraries were prepared following the manufacturer’s protocol (NEBNext Ultra II FS DNA library prep kit). WGS was performed using a NextSeq500 sequencer (Illumina) in paired-end reads (2× 75 nucleotides) or a Novaseq (2× 150 nucleotides). Reads were processed with Cutadapt 2.10^36^ and Trimmomatic 0.39^37^. De novo assembly was performed using Shovill 1.1.0 (RRID:*SCR_017077*). All sequences are available under the NCBI bioproject PRJEB79574^35^ and PRJNA566319 (Tables S1and S2).

Multilocus Sequence Typing (MLST), based on the Achtman scheme, was done with mlst 2.19.0 (https://github.com/tseemann/mlst), and Clermont Typing was performed with the in silico method^38^. Resfinder 4^39^ and Pointfinder^40^ were performed via ABRicate 1.0.1 to detect resistance genes and mutations^41^. Virulence genes were screened using ABRicate 1.2.0 with the vf_coli database, updated on 2026-Jan-28. Search was refined by expert viewing for loci with missing gene, like the *pap* operon, with the identification of a variant expressing a protein annotated as “PapG chaperone-binding domain” (refseq_Protein WP_225092971.1). The *fanCDEFGH* genes (F5 pili biosynthesis) seem misidentified in the vf_coli database, and we instead used the FanCDEFGH protein sequences from Uniprot (P18103, P12050, P25402, P25448, P20861, P20862) in our analyses. Draft genomes were annotated using Prokka 1.14.5^42^. Core-genome MLST (cgMLST) was performed with chewBBACA 3.3.6^43^, based on the RIDOM *E. coli* scheme. The number of alleles different between two isolates was determined with cgMLST-dists 0.4.0 (https://github.com/tseemann/cgmlst-dists).

### Phylogenetic analyses

Maximum likelihood core genome phylogeny of the 764 isolates was determined with Roary, 3.13.0^44^ and RAxML 8.2.12^45^, with *Escherichia fergusonii* (NZ_CP057657) as outgroup. Genomes from the main sequence types (STs) were contextualized with EnteroBase datasets downloaded in July 2022^15^. Only the 1521 ST117 sequences with complete metadata were retained. A preliminary analysis showed that 72 out of the 81 ST10 isolates belong to the cgMLST hierarchical cluster HC100 1126. We included only the 353 genomes from this HC100 out of the 13592 ST10 EnteroBase isolates. ML phylogenies were generated from recombination-filtered core alignments with RAxML^45^, using Snippy v4.6.0 (https://github.com/tseemann/snippy) and Gubbins v2.4.1^46^ and visualized with iTOL v7^47^. The ancestral state (bovine or human) at each node of phylogenies was inferred using PastML v1.9.51 using the MPPA (Maximum Parsimony with Posterior Averaging) prediction method and the F81 model^20^. ML phylogenies for the PastML analysis were inferred with IQ-TREE/2.4.0, using the previous alignments and the GTR + F + ASC + R2 model^20^. Branch support was assessed via 1000 ultrafast bootstrap replicates, and topologies refined by BNNI^48^.

### Geographical distribution of the isolates and clonal dissemination

Isolates and farms were localized according to the GPS coordinates of their postal codes (Open Data Wallonie-Bruxelles, https://www.odwb.be/). Farms were classified as east, west, or crossed by the Meuse. After deduplicating clonal isolates from the same animal, we calculated genetic (cgMLST allelic) and geographic distances between pairs. Maps and network visualizations were generated in R using standard spatial packages (R Core Team, https://www.r-project.org/).

### Statistical analyses

Statistical analyses of isolate characteristics were performed with R and Python3 using Fisher’s exact test or the chi-squared test, as appropriate. The difference in the ARG number between *mcr*-carrying and non-*mcr*-carrying isolates was assessed using the non-parametric Wilcoxon rank-sum test for independent samples.

### Ethics approval

The samples and the results were obtained from a routine veterinary diagnostic laboratory (ARSIA). According to Chapter 8: Art.D63 of the Walloon code (Belgium) for animal welfare (https://wallex.wallonie.be/eli/loi-decret/2018/10/04/2018015578/2019/07/01), no animal experiment was performed. Stool samples were collected non-invasively. The in-farm-dead calves arrive at the laboratory to be necropsied, to find an explanation for their deaths (most of the time, a sepsis). Therefore, ethical approval was not required, as the study complied with relevant national guidelines.

## Supplementary information

Supplementary material

Guerin-Table S8

Guerin-Table-S1-Rev

Guerin-Table-S2-Rev

Guerin-Table-S4

Guerin-Table-S5

## Data Availability

Data that support the findings of this study are provided within the manuscript and the associated supplementary materials. More specifically, all sequences are available under the NCBI bioproject PRJEB7957435 and PRJNA566319 (Tables S1, S2); isolate and sequence data are in Table S1, S2 and S4; geographic and genetic distances between isolate pairs are in Table S5; colistin MIC values of 15 *E. coli* isolates are in Table S3.

## Code availability

No specific code was used in this study. References of the software used in bioinformatic analyses and phylogeny are provided within the manuscript.

## References

[1] Denamur, E., Clermont, O., Bonacorsi, S. & Gordon, D. The population genetics of pathogenic *Escherichia coli*. *Nat. Rev. Microbiol.* **19**, 37–54 (2021). 10.1038/s41579-020-0416-x 32826992

[2] Ramos, S. et al. *Escherichia coli* as commensal and pathogenic bacteria among food-producing animals: health implications of extended spectrum β-lactamase (ESBL) production. *Animals* **10**. 10.3390/ani10122239 (2020). PMC7761174 33260303

[3] Kolenda, R., Burdukiewicz, M. & Schierack, P. A systematic review and meta-analysis of the epidemiology of pathogenic *Escherichia coli* of calves and the role of calves as reservoirs for human pathogenic *E. coli*. *Front. Cell. Infect. Microbiol.* **5**, 23 (2015). 10.3389/fcimb.2015.00023 PMC4357325 25815276

[4] Cho, Y. I. & Yoon, K. J. An overview of calf diarrhea-infectious etiology, diagnosis, and intervention. *J. Vet. Sci.* **15**, 1–17 (2014). 10.4142/jvs.2014.15.1.1 PMC3973752 24378583

[5] Uyama, T. et al. Associations of calf management practices with antimicrobial use in Canadian dairy calves. *J. Dairy Sci.* **105**, 9084–9097 (2022). 10.3168/jds.2022-22299 36175237

[6] Miltgen, G. et al. Circulation of extended-spectrum β-lactamase and plasmid-borne cephalosporinase-producing *Escherichia coli* from a One Health perspective: a narrative review. *Clin. Microbiol. Infect.* **32**, 578–590 (2026). 10.1016/j.cmi.2025.12.003 41380801

[7] Jesudason, T. WHO publishes list of medically important antimicrobials. *Lancet Infect. Dis.* **24**, e284 (2024). 10.1016/S1473-3099(24)00248-2 38677312

[8] Aidara-Kane, A. et al. World Health Organization (WHO) guidelines on use of medically important antimicrobials in food-producing animals. *Antimicrob. Resist. Infect. Control* **7**, 7 (2018). 10.1186/s13756-017-0294-9 PMC5772708 29375825

[9] Tiseo, K., Huber, L., Gilbert, M., Robinson, T. P. & Van Boeckel, T. P. Global trends in antimicrobial use in food animals from 2017 to 2030. *Antibiotics* **9**. 10.3390/antibiotics9120918 (2020). PMC7766021 33348801

[10] Royal Decree of Belgium. *Arrêté Royal du 21 Juillet 2016. Arrêté Royal Relatif aux Conditions d’Utilisation des Médicaments par les Médecins Vétérinaires et par les Responsables des Animaux*. Available online: http://www.etaamb.be/fr/arrete-royal-du-21-juillet-2016_n2016024152.html. (in French) (2016).

[11] Guérin, V. et al. Seven-year evolution of β-lactam resistance phenotypes in Escherichia coli isolated from young diarrheic and septicaemic calves in Belgium. *Vet. Sci*. **9**. 10.3390/vetsci9020045 (2022). PMC8880553 35202298

[12] Andersson, D. I. & Hughes, D. Selection and transmission of antibiotic-resistant bacteria. *Microbiol. Spectr*. **5**. 10.1128/microbiolspec.MTBP-0013-2016 (2017). PMC11687535 28752817

[13] Croucher, N. J. & Didelot, X. The application of genomics to tracing bacterial pathogen transmission. *Curr. Opin. Microbiol* **23**, 62–67 (2015). 10.1016/j.mib.2014.11.004 25461574

[14] Guérin, V. et al. A three-year evolution and comparison of the bla(CTX-M) genes in pathogenic and non-pathogenic *Escherichia coli* isolated from young diarrheic and septicaemic calves in Belgium. *Res. Vet. Sci.* **152**, 647–650 (2022). 10.1016/j.rvsc.2022.09.037 36215736

[15] Zhou, Z., Alikhan, N. F., Mohamed, K., Fan, Y. & Achtman, M. The EnteroBase user’s guide, with case studies on *Salmonella* transmissions, *Yersinia pestis* phylogeny, and *Escherichia* core genomic diversity. *Genome Res.* **30**, 138–152 (2020). 10.1101/gr.251678.119 PMC6961584 31809257

[16] Clermont, O. et al. Characterization and rapid identification of phylogroup G in *Escherichia coli*, a lineage with high virulence and antibiotic resistance potential. *Environ. Microbiol.* **21**, 3107–3117 (2019). 10.1111/1462-2920.14713 31188527

[17] Caroff, N., Espaze, E., Gautreau, D., Richet, H. & Reynaud, A. Analysis of the effects of -42 and -32 *ampC* promoter mutations in clinical isolates of *Escherichia coli* hyperproducing *ampC*. *J. Antimicrob. Chemother.* **45**, 783–788 (2000). 10.1093/jac/45.6.783 10837430

[18] Mulvey, M. R. et al. Molecular characterization of cefoxitin-resistant *Escherichia coli* from Canadian hospitals. *Antimicrob. Agents Chemother.* **49**, 358–365 (2005). 10.1128/AAC.49.1.358-365.2005 PMC538860 15616316

[19] Kieffer, N. et al. MCR-9, an inducible gene encoding an acquired phosphoethanolamine transferase in *Escherichia coli*, and its origin. *Antimicrob. Agents Chemother*. **63**. 10.1128/aac.00965-19 (2019). PMC6709461 31209009

[20] Ishikawa, S. A., Zhukova, A., Iwasaki, W. & Gascuel, O. A fast likelihood method to reconstruct and visualize ancestral scenarios. *Mol. Biol. Evol.* **36**, 2069–2085 (2019). 10.1093/molbev/msz131 PMC6735705 31127303

[21] Lane, M. C. & Mobley, H. L. Role of P-fimbrial-mediated adherence in pyelonephritis and persistence of uropathogenic *Escherichia coli* (UPEC) in the mammalian kidney. *Kidney Int.* **72**, 19–25 (2007). 10.1038/sj.ki.5002230 17396114

[22] Keller, R. et al. Afa, a diffuse adherence fibrillar adhesin associated with enteropathogenic *Escherichia coli*. *Infect. Immun.* **70**, 2681–2689 (2002). 10.1128/IAI.70.5.2681-2689.2002 PMC127892 11953412

[23] Sukalić, T. et al. Impact of virulence genes and pathotypes of intestinal pathogenic *Escherichia coli* on gastrointestinal lesions in pre- and post-weaning piglets. *Front. Cell. Infect. Microbiol.* **15**, 1704407 (2025). 10.3389/fcimb.2025.1704407 PMC12883757 41668732

[24] *AMCRA. Activités et Réalisations Relatives à la Réduction de l’usage d’antibiotiques et de l’antibiorésistance chez les animaux en Belgique 2021–2024*. Available online https://www.amcra.be/swfiles/files/Publiek-rapport-2024_FRANS_Finaal.pdf (In French, 2025).

[25] Haenni, M., Châtre, P. & Madec, J. Y. Emergence of *Escherichia coli* producing extended-spectrum AmpC β-lactamases (ESAC) in animals. *Front. Microbiol.* **5**, 53 (2014). 10.3389/fmicb.2014.00053 PMC3924575 24592257

[26] Alzayn, M. et al. Characterization of AmpC-hyperproducing *Escherichia coli* from humans and dairy farms collected in parallel in the same geographical region. *J. Antimicrob. Chemother.* **75**, 2471–2479 (2020). 10.1093/jac/dkaa207 32542329

[27] Xia, F. et al. Complete genomic analysis of ST117 lineage extraintestinal pathogenic *Escherichia coli* (ExPEC) to reveal multiple genetic determinants to drive its global transmission: ST117 *E. coli* as an emerging multidrug-resistant foodborne ExPEC with zoonotic potential. *Transbound. Emerg. Dis.* 10.1111/tbed.14678 (2022). 35945191

[28] Ronco, T. et al. Spread of avian pathogenic *Escherichia coli* ST117 O78:H4 in Nordic broiler production. *BMC Genomics* **18**, 13 (2017). 10.1186/s12864-016-3415-6 PMC5210278 28049430

[29] Celagri. [Key figures] *The Dairy Calf Fattening Sector*(*in French [Chiffres clés] La filière d’engraissement des Veaux Laitiers*) http://www.celagri.be/wp-content/uploads/2024/02/La-filiere-veau-chiffres-cles-2022.pdf (2024).

[30] Wetzel, A. N. & LeJeune, J. T. Clonal dissemination of *Escherichia coli* O157:H7 subtypes among dairy farms in northeast Ohio. *Appl. Environ. Microbiol.* **72**, 2621–2626 (2006). 10.1128/AEM.72.4.2621-2626.2006 PMC1449053 16597966

[31] *Celagri. Cheptel Bovin Laitier-État de l’Agriculture Wallonne (Dairy Cattle Herd-State of Walloon Agriculture)*. Available online https://www.file:///Users/pglaser/Downloads/Bovins_laitiers.pdf (In French, 2024).

[32] *Celagri. C**heptel Bovin Viandeux-Etat de l’Agriculture Wallonne (Beef Cattle Herd-State of Walloon Agriculture)*. Available online: http://www.file:///Users/pglaser/Downloads/Bovins_viandeux.pdf (In French, 2024).

[33] Ellis, J., Brown, E., Colenutt, C., Schley, D. & Gubbins, S. Inferring transmission routes for foot-and-mouth disease virus within a cattle herd using approximate Bayesian computation. *Epidemics* **46**, 100740 (2024). 10.1016/j.epidem.2024.100740 38232411

[34] CASFM-vet. *C**omité de l’antibiogramme de la Société Française de Microbiologie RECOMMANDATIONS VÉTÉRINAIRES*. https://www.sfm-microbiologie.org/wp-content/uploads/2023/06/CASFM_VET2023.pdf. (2023).

[35] Guérin, V. et al. Identification of β-lactamase-encoding (bla) genes in phenotypically β-lactam-resistant *Escherichia coli* isolated from young calves in Belgium. *Microb. Drug Resist.* **27**, 1578–1584 (2021). 10.1089/mdr.2020.0472 33913753

[36] Martin, M. Cutadapt removes adapter sequences from high-throughput sequencing reads. *EMBnet J*. **10**, 10.14806/ej.17.1.200 (2011).

[37] Bolger, A. M., Lohse, M. & Usadel, B. Trimmomatic: a flexible trimmer for Illumina sequence data. *Bioinformatics* **30**, 2114–2120 (2014). 10.1093/bioinformatics/btu170 PMC4103590 24695404

[38] Beghain, J., Bridier-Nahmias, A., Le Nagard, H., Denamur, E. & Clermont, O. Clermonyping: an easy-to-use and accurate in silico method for Escherichia genus strain phylotyping. *Microbial. Genom*. **4**. 10.1099/mgen.0.000192 (2018). PMC6113867 29916797

[39] Bortolaia, V. et al. ResFinder 4.0 for predictions of phenotypes from genotypes. *J. Antimicrob. Chemother.* 10.1093/jac/dkaa345 (2020). PMC7662176 32780112

[40] Zankari, E. et al. PointFinder: a novel web tool for WGS-based detection of antimicrobial resistance associated with chromosomal point mutations in bacterial pathogens. *J. Antimicrob. Chemother.* **72**, 2764–2768 (2017). 10.1093/jac/dkx217 PMC5890747 29091202

[41] Zankari, E. et al. Identification of acquired antimicrobial resistance genes. *J. Antimicrob. Chemother.* **67**, 2640–2644 (2012). 10.1093/jac/dks261 PMC3468078 22782487

[42] Seemann, T. Prokka: rapid prokaryotic genome annotation. *Bioinformatics* **30**, 2068–2069 (2014). 10.1093/bioinformatics/btu153 24642063

[43] Silva, M. et al. chewBBACA: a complete suite for gene-by-gene schema creation and strain identification. *Microb. Genom*. **4**. 10.1099/mgen.0.000166 (2018). PMC5885018 29543149

[44] Page, A. J. et al. Roary: rapid large-scale prokaryote pan genome analysis. *Bioinformatics* **31**, 3691–3693 (2015). 10.1093/bioinformatics/btv421 PMC4817141 26198102

[45] Stamatakis, A. RAxML version 8: a tool for phylogenetic analysis and post-analysis of large phylogenies. *Bioinformatics* **30**, 1312–1313 (2014). 10.1093/bioinformatics/btu033 PMC3998144 24451623

[46] Croucher, N. J. et al. Rapid phylogenetic analysis of large samples of recombinant bacterial whole genome sequences using Gubbins. *Nucleic Acids Res.* **43**, e15 (2015). 10.1093/nar/gku1196 PMC4330336 25414349

[47] Letunic, I. & Bork, P. Interactive Tree Of Life (iTOL) v5: an online tool for phylogenetic tree display and annotation. *Nucleic Acids Res.* **49**, W293–w296 (2021). 10.1093/nar/gkab301 PMC8265157 33885785

[48] Minh, B. Q. et al. IQ-TREE 2: new models and efficient methods for phylogenetic inference in the genomic era. *Mol. Biol. Evol.* **37**, 1530–1534 (2020). 10.1093/molbev/msaa015 PMC7182206 32011700

