# Supplementary material for: Antibiotic resistance and virulence shape the population of E. coli from diseased calves in Wallonia, Belgium
Source: NPJ Antimicrob Resist. 2026 Jun 25;4:71. doi: 10.1038/s44259-026-00235-2 (PMC13745570; doi:10.1038/s44259-026-00235-2)
Supplement: Supplementary file 1 [file 44259_2026_235_MOESM1_ESM.pdf]

Antibiotic resistance and virulence shape the population of *E. coli* from diseased calves in Wallonia, Belgium.

By Virginie Guérin, Nicolas Cabanel, Gan Min Marissa Diana Meijer, Guilhem Royer, Jean-Noël Duprez, Jacques G. Mainil, Marc Saulmont, Damien Thiry, Philippe Glaser.

Supplementary material.

Supplementary results.

Comparison of virulence genes detection and agglutination assay. Isolates were tested by agglutination assay for three antigens: the F5 (K99) and F17 fimbriae and the adhesin CS31A. The F5 fimbriae is encoded by the *fanCDEFGH* operon[1], which was present in 18 isolates and all except one was identified as F5 positive by agglutination. The 17 F5 positive isolates carried the *fan* operon. Among the isolates carrying a partial or a complete *clp* operon (CS31A biogenesis[2]), 27 were negative for the three antigens tested, 14 were positive for F5 (showing a partial *clp* locus) and three positive for F17. Conversely, in 48 isolates positive for the CS31A antigen, none of the *clp* gene were detected. There are several loci-variants for F17 biogenesis[3]. An *f17* locus (complete or partial) was detected in only 62 (56%) out of the 109 isolates positive for F17. Note that 35 of the 47 F17 positive isolates in which an *f17* locus was not detected belonged to the lineage L-ST88-3. These isolates might express an antigen cross-reacting in the agglutination assay and missing from the vf_coli database. Conversely eight and 19 isolates carrying a complete or a partial *f17* locus respectively were negative in the agglutination assay. Discrepancies between the genomic detection and the phenotypic assay might result of experimental limitations, the absence of expression under the condition tested or a defective operon and of the lack of genetic determinants in the vf_coli database.

Table S1: Characteristics of *E. coli* isolates collected during the three calving seasons, S1, S2 and S3. Excel file Table S1

§age: 0 means the first day of life; &side: East or West of the Meuse, Meuse indicates that the Meuse crosses the postal code; #Other isolate from the same animal: no, means that there is no other isolate from the same animal in our collection, in bold sequenced isolate, a star indicates that the isolates from this animal are clonal (less than five different alleles by cgMLST), $P/NP: P is for pathogenic and NP for non-pathogenic as defined in the main text. ∂ND stand for not determined €Minca: the positive antigen is indicated; N for negative. ßEhly negative (N) or positive (P) for Enterohemolysin activity, H+ is for alpha-hemolysin; *McConkey-CTX: positive (P) or negative (N) for growth on MacConkey plate supplemented with 1 mg/L cefotaxime; %Lineage: details on lineages are in Table 2. £Antibiotic susceptibility testing, inhibition zone diameter and Susceptible (S), Intermediate (I), or Resistant (R) phenotype according to EUCAST breakpoints. ND, not determined. AMX, Amoxicillin, AMC, Amoxicillin / Clavulanate; XNL, Ceftiofur; CFQ, Cefquinome, CTX, Cefotaxime; CTC, Cefotaxime / Clavulanate; FOX, Cefoxitin; MER, Meropenem. ## Number of classes based on gene and mutation detection.

Table S2: Characteristics of *E. coli* isolates collected during the two calving seasons, aS1 and aS2 Excel file Table S2

&side: East or West of the Meuse. Meuse indicates that the Meuse crosses the postal code; #Other isolate from the same animal: no, means that there is no other isolate from the same animal in our collection. $P/NP: P is for pathogenic and NP for non-pathogenic as defined in the main text; €Minca: the positive antigen is indicated; N for negative. ßEhly negative (N) Enterohemolysin activity; ∂ND stand for not determined; %Lineage: details on lineages are in Table 2. ## Number of classes based on gene and mutation detection.

Table S3. MIC of isolates carrying *mcr* genes

Isolate	*mcr* gene	&MIC colistin (ng/µl)
MG1655	-	0.5 - 0.5
R0042	*mcr-1*	4 - 4
R0238	*mcr-1*	4 - 4
R0301	*mcr-1*	8 - 8
R0506	*mcr-2*	32 - 32
R1211	*mcr-1*	4 - 8
R1466	*mcr-2*	16 - 4
R1589	*mcr-9*	0.5 - 1
R0027	*mcr-1*	8 - 4
R0050	*mcr-1*	4 - 4
R0053	*mcr-1*	4 - 4
R0235	*mcr-1*	4 - 4
R0923	*mcr-1*	4 - 4
R1127	*mcr-1*	4 - 4
R0107	*mcr-9*	0.5 - 4

&MIC were determined in by microdilution using Thermofisher Sensititre plates. Independent duplicates were performed.

Table S4: **Characteristics of *E. coli* isolates from EnteroBase clustering with isolates from the study**. Excel file Table S4

§ID Enterobase identifiers. Annotations were retrieved from Enterobase. %Lineage: details on lineages are in Table 2. ## Number of classes based on gene and mutation detection.

Table S5: Genetic and geographic distances of isolates pairs with less than 21 different cgMLST alleles. Excel file Table S5

[&]Genetic distances in number of different cgMLST alleles. [§]ST is for Sequence Type of each isolate. [#]Position relative to the Meuse River. Meuse means that the postal code is crossed by the Meuse.

Table S6 Geographical and phenotypic distribution of *E. coli* isolates

	Strain collection (S1-3)					Deduplicated sequenced isolates (aS1-2, S1-3)				
[§]Localization	All strains	NSBL	ESBL	AmpC-like	AmpC	All strains	AmpC-like	ST88	ST88-Cluster (%ST88, % all)	ST88 other
East	813	449 (55,2%)	184 (22,6%)	147 (18%)	29 (3,6%)	415	128	148 (18,1%)	126 (85%, 15,4%)	22 (2,7%)
West	654	375 (57,3%)	195 (29,8%)	59 (9%)	21 (3,2%)	306	51	55 (8,4%)	37 (67,3%, 5,7%)	18 2,8%
Meuse	26	13 (50%)	8 (30,8%)	5 (19,2%)	0	15	5	4 (15,4%)	2 (50%, 7.7%)	2 (7.7%)
P value[&]		0.43	[@]0.0022	<0.0001	0.67		<0.0001	<0.0001	0.0089 (ST88)<br><0.0001 (all)	[*]0.089 (ST88)<br>0,74 (all)

[§]Relative to Meuse River; Meuse is for herds in postal codes crossed by the Meuse. [&]Two-tailed exact Fisher test, East compared to West. [@]P value that ESBL are more frequent on the West compared to the East. [*]P value that ST88 are more frequent outside cluster on the west compared to the east.

Table S7 Isolate pairs with less than 21 different alleles and isolates with at least one match

	[#]All strains	[§]P values	[#@]AmpC-Like	[§]P values	[#]No AmpC-Like	[§]P values	[#]ST88	[§]P value	[#]No ST88	[§]P valuer
[€]East <=> East	2130 (281)	-	1875 (110)	-	255 (171)	-	1907 (124)		223 (157)	
[€]West <=> West	131 (141)	[$]< 0.0001	40 (35)	[$]0.011	91 (106)	[$]<0.0001	35 (28)	[$]<0.0001	96 (113)	[$]0.0021
[%€]East <=> West	263 (203)	[$]0.0014<br>[£]0.0083	44 (38)	[$]<0.0001<br>[£]0.0047	219 (165)	[$]0.8<br>[£]<0.0001	48 (47)	[$]<0.0001<br>[£]0.62	215 (156)	[$]0.79<br>[£]0.0007
Intra-farm East	49 (65)									
Intra-farm West	34 (41)	[*]0.4566								

[#]Number of isolate pairs with less than 21 different alleles by cgMLST and in parenthesis, numbers of isolates with at least one match. [§]Two-tailed exact Fisher test on the proportion of isolates with at least one match. [@]At least one of the two isolates showed an AmpC-like phenotype. [€]Excluding intra-farm pairs. [%]For isolates pairs on both sides of the Meuse, the average of the number of isolates in each compartment was used for statistical analyses. [$]Compared with East-East. [£]compared with West-West. *Compared with Intra-farm East.

Table S8. Virulence factors identified among the 764 isolates form sick calves. Excel file Table S8

[#]Other isolate from the same animal: no, means that there is no other isolate from the same animal in our collection, in bold sequenced isolate, a star indicates that the isolates from this animal are clonal (less than five different alleles by cgMLST). [%]Lineage: details on lineages are in Table 2. [&]side: East or West of the Meuse, Meuse indicates that the Meuse crosses the postal code;, [$]P/NP: P is for pathogenic and NP for non-pathogenic as defined in the main text. [∂]ND stand for not determined [€]Minca: the positive antigen is indicated; N for negative. [ß]Ehly negative (N) or positive (P) for Enterohemolysin activity, H+ is for alpha-hemolysin; *McConkey-CTX: positive (P) or negative (N) for growth on MacConkey plate supplemented with 1 mg/L cefotaxime. [£]Partial indicates that not all the genes of the locus were identified. The locus might be partial, or gene variants were not detected (missing in the vf_database). ##Variant indicates that a *papG* like gene was detected instead of a *papG* allele.

Table S9 Occurrence of virulence factors (in %) in *E. coli* isolates from sick calves and from EnteroBase

Phylogroup	ST	Collection	Source	Count by source	astA	afa		CS31A		F17		pap			sit		yersiniabactin		salmochelin		aérobactin	
					present	[&]partial	present	partial	present	partial	present	partial	present	variant	partial	present	partial	present	partial	present	partial	present
A	10	ARSIA-RUBLA	Livestock	81	12.3	3.7	7.4	19.8	3.7	1.2	7.4	0	0	17.3	8.6	66.7	0	2.5	3.7	14.8	4.9	63
		Enterobase	Livestock	98	21.4	14.3	9.2	22.4	6.1	0	6.1	2	1	17.3	3.1	39.8	0	10.2	5.1	7.1	0	38.8
			Human	63	3.2	1.6	3.2	1.6	0	0	0	0	4.8	12.7	12.7	42.9	3.2	30.2	1.6	17.5	3.2	34.9
	167	ARSIA-RUBLA	Livestock	45	20	2.2	17.8	0	6.7	51.1	8.9	0	0	51.1	2.2	91.1	2.2	31.1	4.4	2.2	17.8	71.1
		Enterobase	Livestock	30	3.3	33.3	3.3	0	0	6.7	20	0	0	23.3	0	76.7	0	26.7	0	16.7	0	83.3
			Human	512	7.2	2.1	0	0.2	0.2	0.4	0.4	0	2	1	0	25.8	0.6	44.3	0	4.5	0.2	28.5
	617	ARSIA-RUBLA	Livestock	11	0	0	63.6	0	90.9	0	0	0	0	18.2	0	81.8	0	100	0	0	0	81.8
		Enterobase	Livestock	26	26.9	34.6	3.8	0	3.8	0	0	0	0	19.2	3.8	38.5	0	53.8	3.8	7.7	3.8	38.5
			Human	244	14.8	0.8	0.4	0	0	0	0	0	5.7	2.5	0	67.6	0	72.1	0	5.7	0	64.8
	744	ARSIA-RUBLA	Livestock	16	0	0	0	0	12.5	25	0	0	0	0	12.5	37.5	0	0	0	12.5	0	50
		Enterobase	Livestock	260	16.2	1.2	1.5	0.4	1.9	1.5	0	0	0	2.3	0.8	17.3	0	1.5	0.8	6.2	0.4	16.9
			Human	256	8.2	0.4	0.8	0	0	1.2	0.4	0	0	1.2	0	39.8	0	3.9	0.4	19.1	0.4	36.7
	2325	ARSIA-RUBLA	Livestock	17	0	0	0	0	0	0	0	0	0	0	41.2	58.8	0	0	0	0	0	82.4
		Enterobase	Livestock	39	0	0	0	0	0	0	0	0	0	0	0	97.4	0	2.6	0	0	0	2.6
			Human	5	20	0	0	0	0	0	0	0	0	0	0	60	0	20	0	0	0	0
B1	58	ARSIA-RUBLA	Livestock	18	11.1	22.2	11.1	0	0	16.7	5.6	0	5.6	38.9	11.1	72.2	0	72.2	0	83.3	0	83.3
		Enterobase	Livestock	443	14.2	7.2	2.7	1.1	0.7	11.5	2.7	2.9	4.1	3.4	0.7	45.8	2.3	42.4	1.1	41.3	0.2	46.5
			Human	140	9.3	3.6	7.1	1.4	0	2.1	0	3.6	2.1	7.9	4.3	67.1	5.7	55.7	0	57.9	0	69.3
	1642	ARSIA-RUBLA	Livestock	14	0	7.1	0	0	0	0	21.4	7.1	0	7.1	7.1	92.9	0	0	0	0	7.1	92.9
		Enterobase	Livestock	24	12.5	0	0	4.2	0	12.5	0	0	0	0	0	25	0	4.2	0	0	0	25
			Human	7	0	0	0	0	0	0	0	0	0	14.3	0	42.9	0	0	0	0	0	28.6
C	88	ARSIA-RUBLA	Livestock	222	52.7	42.3	31.5	0	29.3	0.9	3.2	0.5	5.9	66.7	21.2	78.4	2.7	89.6	1.8	30.2	7.2	91.9
		Enterobase	Livestock	426	26.1	16.9	13.8	0.5	15	3.8	0.7	0.9	11	29.1	23.7	57.5	1.9	74.2	2.3	49.5	0.7	77
			Human	268	17.5	17.2	20.1	0	4.9	1.5	0.4	0	7.5	30.2	18.3	63.1	4.9	72.8	2.2	57.1	1.1	70.5
	783	ARSIA-RUBLA	Livestock	29	6.9	3.4	72.4	0	100	93.1	0	0	0	100	3.4	96.6	0	100	0	0	3.4	96.6
		Enterobase	Livestock	5	40	20	40	0	80	60	0	0	0	60	0	100	0	100	0	20	0	100
			Human	4	0	25	50	0	75	100	0	0	0	50	25	75	25	75	0	25	0	100
D	69	ARSIA-RUBLA	Livestock	36	33.3	13.9	50	0	58.3	13.9	0	2.8	0	33.3	30.6	69.4	8.3	25	2.8	5.6	5.6	86.1
		Enterobase	Livestock	19	5.3	0	15.8	0	31.6	5.3	0	0	10.5	5.3	42.1	57.9	0	42.1	0	26.3	0	47.4
			Human	295	3.1	3.1	1.7	0.3	0.3	1.4	0	12.2	52.9	0.7	12.5	80.7	12.9	66.8	1	8.8	4.4	70.2
	106	ARSIA-RUBLA	Livestock	10	100	0	100	0	90	0	0	0	0	0	10	90	0	0	0	0	0	100
		Enterobase	Livestock	17	29.4	11.8	47.1	0	64.7	11.8	0	0	0	11.8	41.2	47.1	0	0	0	11.8	5.9	58.8
			Human	7	0	0	0	0	0	0	0	0	0	14.3	0	100	0	14.3	0	0	0	0
	362	ARSIA-RUBLA	Livestock	13	76.9	0	69.2	0	61.5	0	0	0	0	61.5	30.8	30.8	7.7	23.1	0	0	0	53.8
		Enterobase	Livestock	41	48.8	4.9	41.5	2.4	31.7	0	0	2.4	0	34.1	7.3	39	9.8	12.2	0	2.4	0	22
			Human	96	55.2	1	14.6	34.4	7.3	0	0	1	2.1	21.9	7.3	64.6	11.5	11.5	0	4.2	1	13.5
E	57	ARSIA-RUBLA	Livestock	13	53.8	0	53.8	0	23.1	0	0	0	0	46.2	61.5	38.5	0	38.5	0	92.3	0	76.9
		Enterobase	Livestock	46	32.6	0	21.7	8.7	15.2	2.2	0	0	15.2	19.6	47.8	41.3	2.2	43.5	8.7	54.3	0	65.2
			Human	47	25.5	0	6.4	34	0	0	0	0	12.8	2.1	29.8	34	2.1	61.7	0	46.8	0	51.1
G	117	ARSIA-RUBLA	Livestock	71	21.1	2.8	16.9	0	97.2	0	0	0	0	78.9	9.9	90.1	4.2	88.7	0	2.8	2.8	97.2
		Enterobase	Livestock	176	17.6	1.7	13.1	0.6	9.1	0	0	0	30.1	12.5	5.7	86.9	4.5	69.3	1.7	61.4	1.1	95.5
			Human	131	23.7	1.5	9.2	0	4.6	0	0	0.8	21.4	8.4	6.1	77.9	2.3	81.7	0.8	71.8	0.8	75.6
			Poultry	800	10.5	0	0.1	0.8	0	0	0	0.2	21.2	1	20.6	76.1	0.4	62.1	3.4	85.8	1.8	88.4
[§]Total of 14 ST		ARSIA-RUBLA	Livestock	596	32.6	18.6	28.5	2.7	37.3	10.9	3.5	0.5	2.4	51.4	16.6	76.3	2.3	58.4	1.7	18.2	5.7	84.1
		Enterobase	Livestock	1650	21.1	13.12	13.4	3.7	15.6	6.1	2.7	1.2	8.9	21.6	14.5	59.8	1.6	50.4	2.1	33.5	0.6	65.5
			Human	2075	13.6	8.5	12.2	1.8	6.2	5.6	0.2	2.1	9.8	19.9	12.2	60	5	57.5	1.2	37.8	1.3	57.6
other ST		ARSIA-RUBLA	Livestock	168	11.3	8.3	17.3	1.8	18.5	7.7	8.9	3.6	3	22.6	8.3	67.9	3.6	28.6	0	11.9	3	64.9

[&]Partial if one or several gene are missing. It could correspond to missing genes or variant not detected by virulence finder. [§]percentage was normalized according to the proportion of isolates among isolates from the study.

Table S10 Resistance and susceptibility characteristic of the four β-lactam resistance profiles

Resistance profiles	AMX	AMC	XNL	CFQ	CTX	CTC	FOX	MER
NSBL	R	S/R	S	S	S	S	S	S
AmpC-like	R	S/R	S	S	S	S	I/R	S
AmpC	R	R	R	R	R	R	R	S
ESBL	R	S/I	R	R	R	S/I	S	S

Amoxicillin, AMX; Amoxicillin / Clavulanate, AMC; Ceftiofur XNL; Cefquinome, CFQ; Cefotaxime, CTX; Cefotaxime / Clavulanate, CTC; Cefoxitin, FOX; Meropenem, MER. R, resistant, S susceptible, I intermediate according to the CASFM-vet guidelines[4]

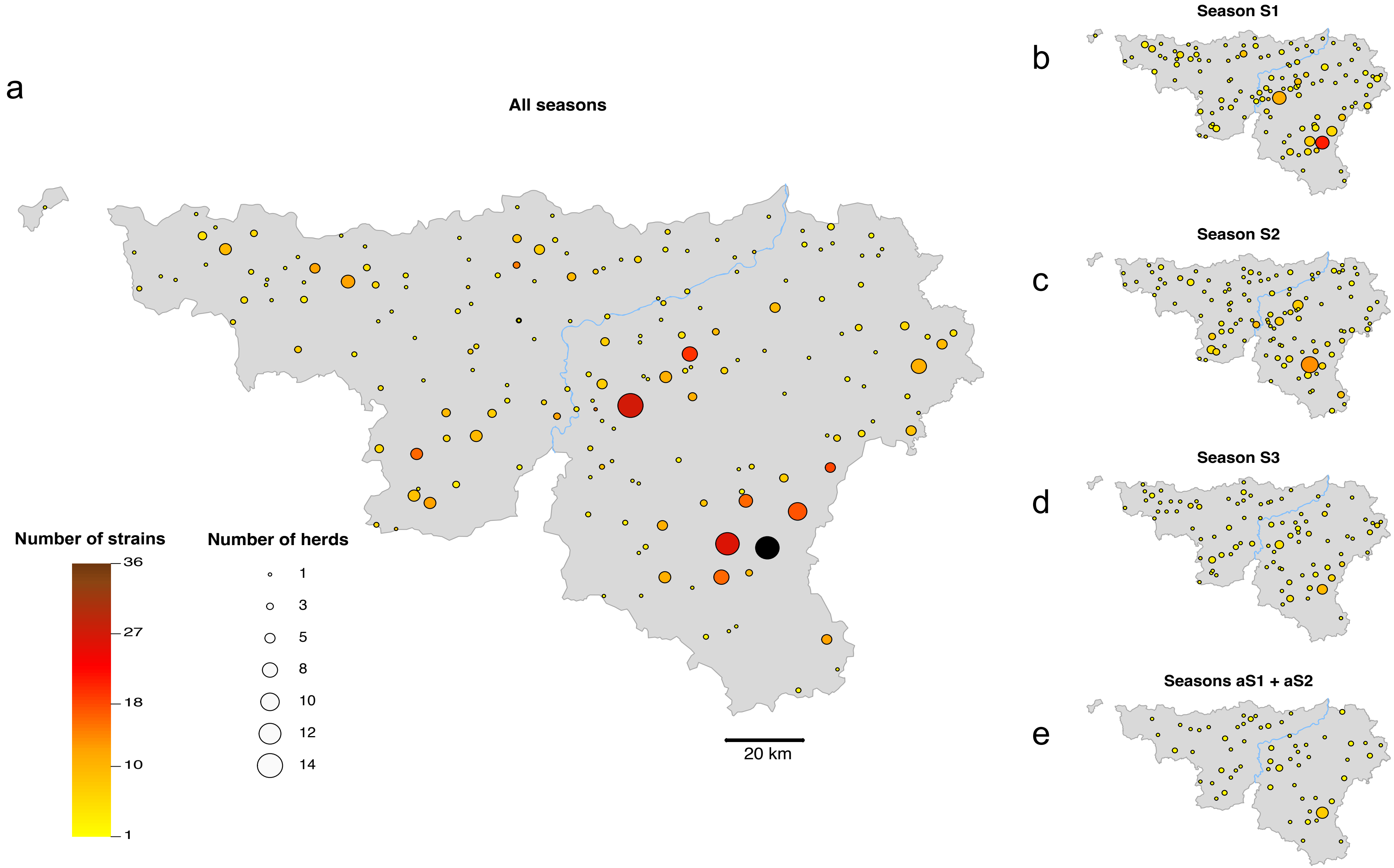

Fig. S1. Geographical distribution of farms and animals across the Walloon territory. Number of farms and animals in each postal code (PC) providing samples are represented by colored disk. Disk were positioned according to the geographical coordinates of the PC (from the Open Data Wallonie-Bruxelles web site https://www.odwb.be/). The size of the disc and its color indicate the number of herds and the number of *E. coli* isolates in each PC, respectively, according to the figure key. **a** isolates from the five calving seasons, **b** season S1, 2017-2018, **c** season S2, 2018-2019, **d** season S3, 2019-2020, **e** season aS1 and aS2, 2015-2017.

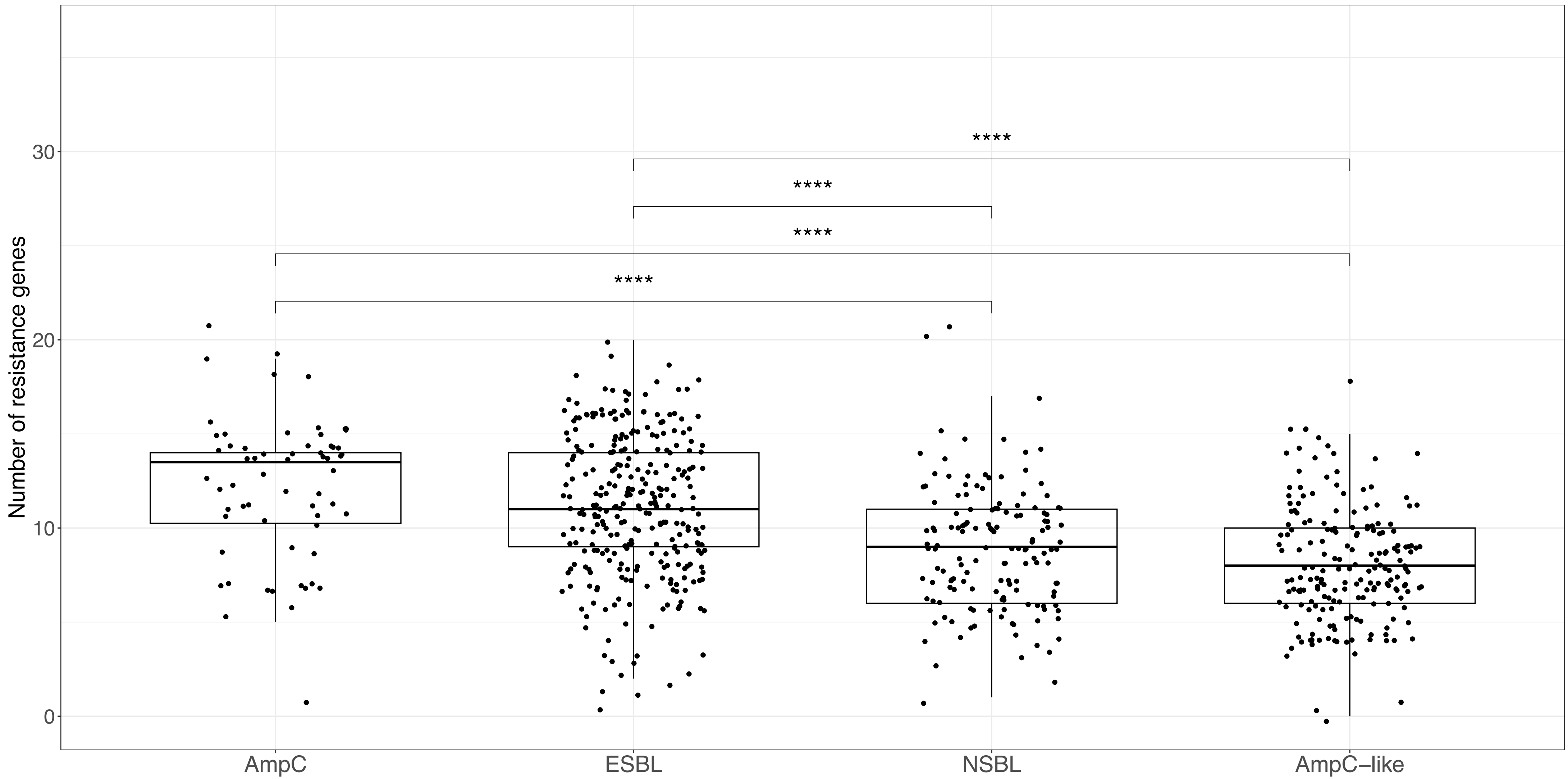

Fig. S2: **Number of acquired resistance genes per isolate in the four β-lactam resistance profiles.** Isolates were from the three calving seasons S1-3. The horizontal lines in the boxes represent the median number of ARGs. The box boundaries represent the first and third quartiles of the distribution and box-plot whiskers span 1.5 times the interquartile range of the distribution. Group differences were first assessed using a global Kruskal-Wallis test, followed by pairwise Dunn's post-hoc tests with Benjamini-Hochberg correction for multiple comparisons. Significant differences were observed between most groups, **** p-value < 0.00005. β-lactam resistance profiles are indicated below the chart.

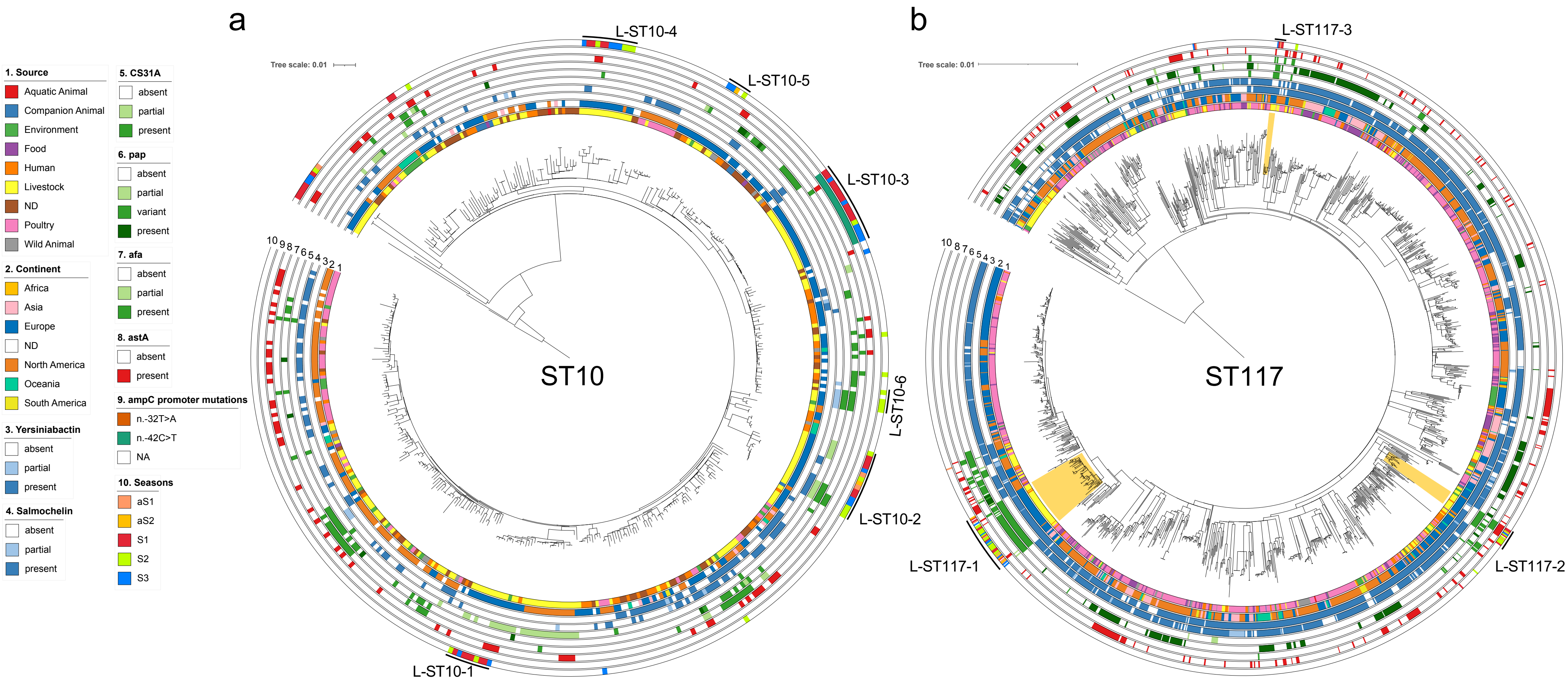

Fig. S3 Phylogeny and characteristics of ST10 and ST117 *E. coli* isolates from the five calving seasons combined with EnteroBase isolates. Maximum likelihood phylogeny was performed by using RAxML 8.2.12 [5] based on recombination-free core genome alignment, computed with Snippy 4.6.0 (https://github.com/tseemann/snippy) and Gubbins 2.4.1 to filter recombined regions[6]. **a.** ST10 (n=81) The tree was built by including 353 ST10 isolates from EnteroBase with a cgMLST distance of less than 100 alleles with strain R0001 (lineage L-ST10-3). Among the 267 known hosts, 71 were bovine, 63 human et 40 poultry. **b.** ST117 (n=71) the tree was built by including only EnteroBase sequences with country and host information (n=1525). 67% Of the isolates were from poultry origin**.** Genomes were annotated according to the figure key as circles from inside to outside: source niche, continent of origin, yersiniabactin, salmochelin, CS31A, pap, afa, astA, P_{ampC} mutation and the season of isolation. The cluster reported in table 2 are indicated outside the last circle.

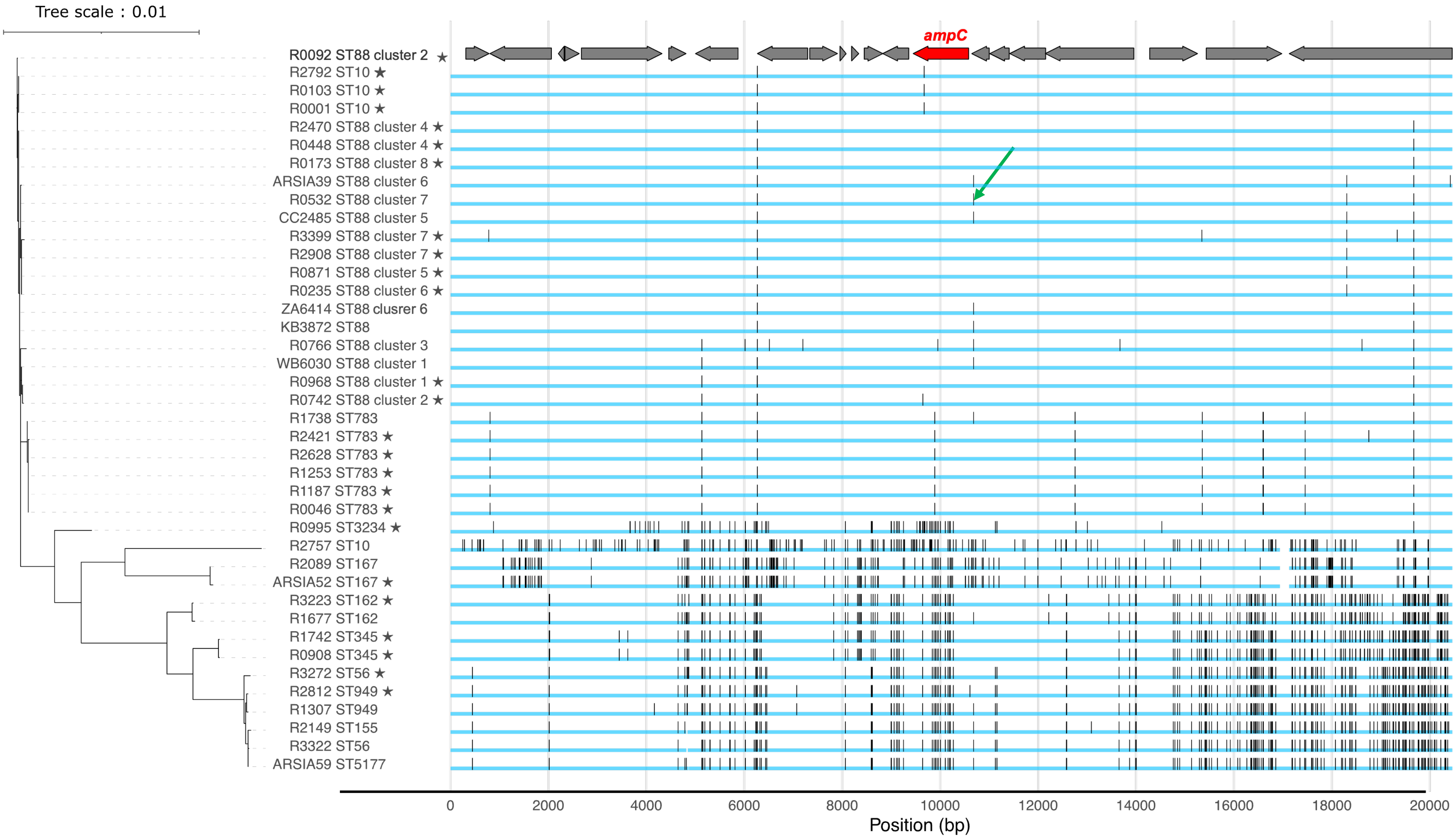

Fig. S4. Recombination detection at the *ampC* locus. SNPs in the chromosomal region 10 kb upstream and downstream *ampC* in 23 strains mutated in P_{ampC} (-42) and in 15 closely related strains non mutated. SNP were identified by using Breseq[7]. Reference sequence was strain R0092 from lineage L-ST88-2 and mutated in P_{ampC}. SNPs are indicated by small vertical lines. Recombination was detected only in the three isolates belonging to the lineage L-ST10-3. The P_{ampC} variant (wild type) is highlighted by a green arrow. The *ampC* gene is shown by a red arrow. Sequences were ordered according to the phylogeny of the visualized *ampC* region shown on the left part.

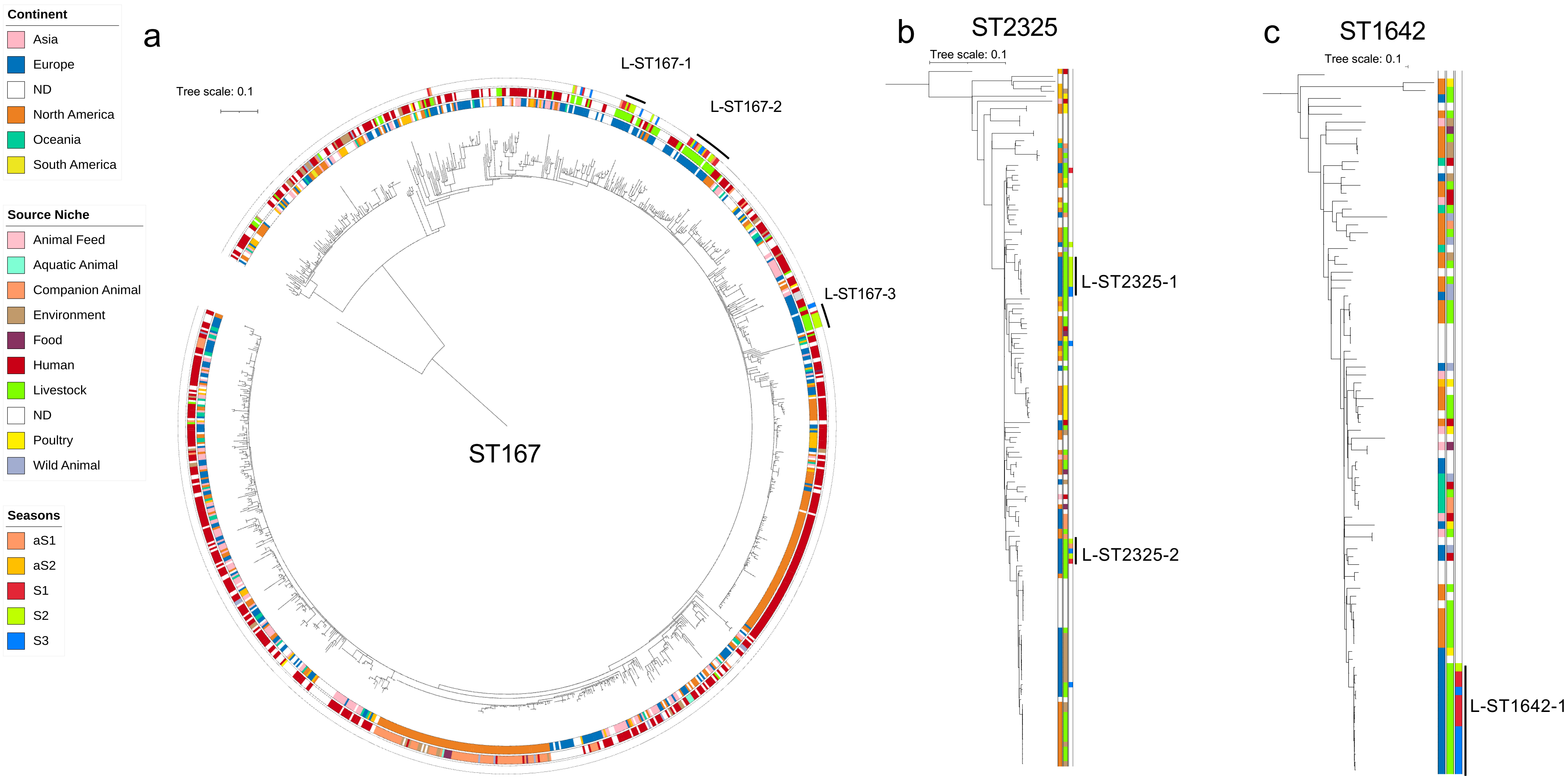

Fig. S5. Phylogeny and characteristics of ST117, ST167, ST2325 and ST1642 *E. coli* isolates from the 5 seasons. ML phylogeny was performed by using RAxML 8.2.12 [5] based on recombination-free core genome alignment, computed with Snippy 4.6.0 and Gubbins 2.4.1 to filter recombined regions[6]. **a**. ST167 (n=44). The tree was built by including 989 ST167 isolates from EnteroBase. Among the 734 with known hosts, 532 were of human origin and 25 of bovine origin. **b**. ST2325 (n=17). 123 strains were retrieved from EnteroBase, with five of human origin and 22 of bovine origin from the 90 with known host. **c**. ST1642 (n=14). 81 isolates were retrieved from EnteroBase (57 annotated), with seven of human origin and 13 of bovine origin. Genome were annotated according to the figure key as circles from inside to outside (a) or from left to right (b and c): continent of origin, source niche, and season of isolation for the isolates of the study. The cluster reported in table 2 are indicated outside the last circle (a) or on the right of the tree (b and c).

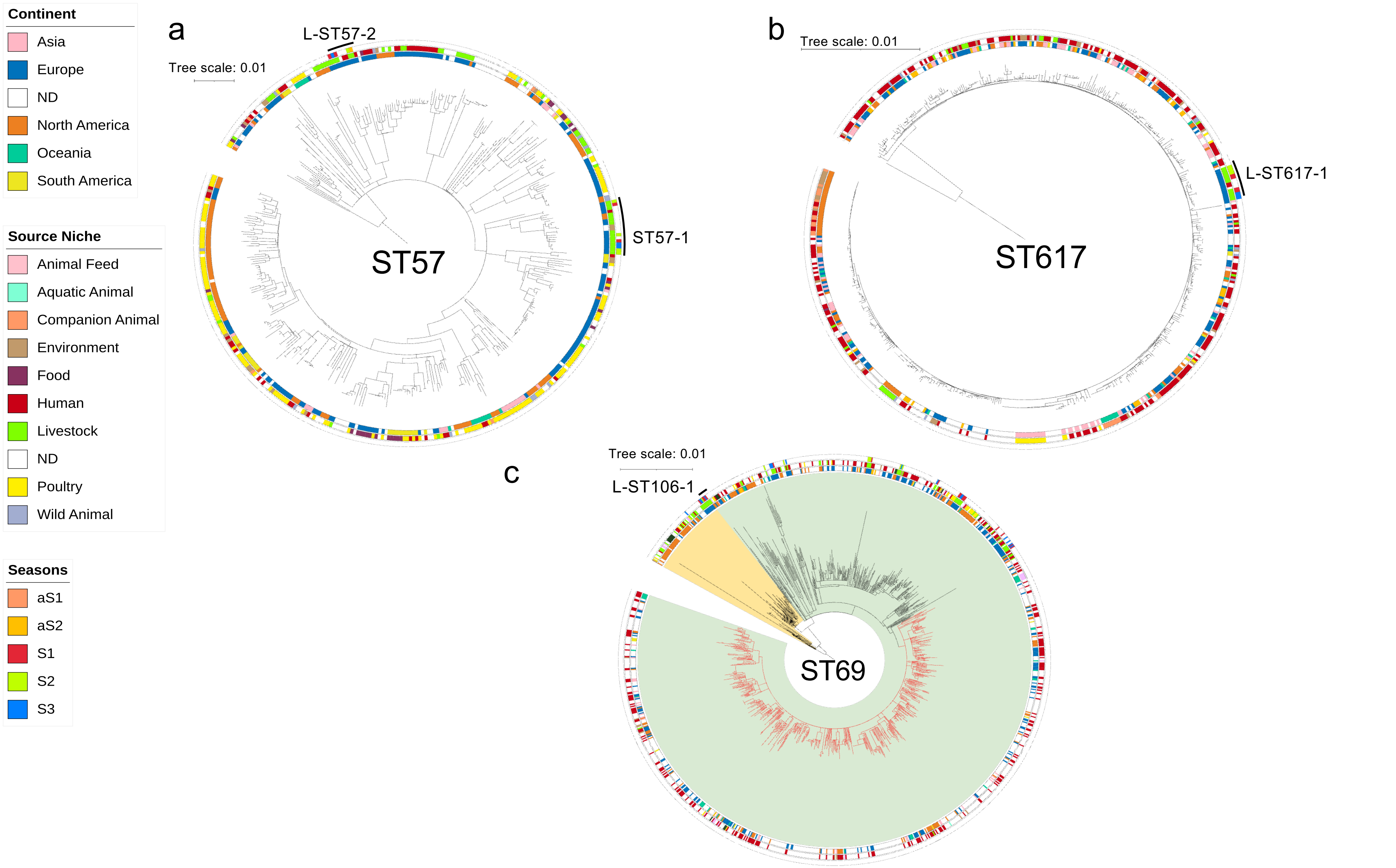

Fig. S6. Phylogeny and characteristics of ST57, ST617 and ST69/ST106 *E. coli* isolates from the 5 seasons combined with EnteroBase isolates. Maximum likelihood phylogeny was performed by using RAxML 8.2.12 [5] based on recombination-free core genome alignment, computed with Snippy 4.6.0 and Gubbins 2.4.1 to filter recombined regions[6]. **a.** ST57, 394 EnteroBase isolates (311 annotated), 50 of human and 20 of bovine origin. **b**, ST617, 561 EnteroBase isolates (386 annotated), 256 of human and 15 of bovine origin. **c**, ST69 (green) / ST106 (yellow), 1103 EnteroBase isolates (467 annotated), 302 of human and 68 of bovine origin. In ST69, note the absence of isolate of the study in the late appearing clade gathering human isolates (in red). Genome were annotated according to the figure key as circles from inside to outside : the continent of origin, the source niche, and the season of isolation. The cluster reported in table 2 are indicated outside the last circle.

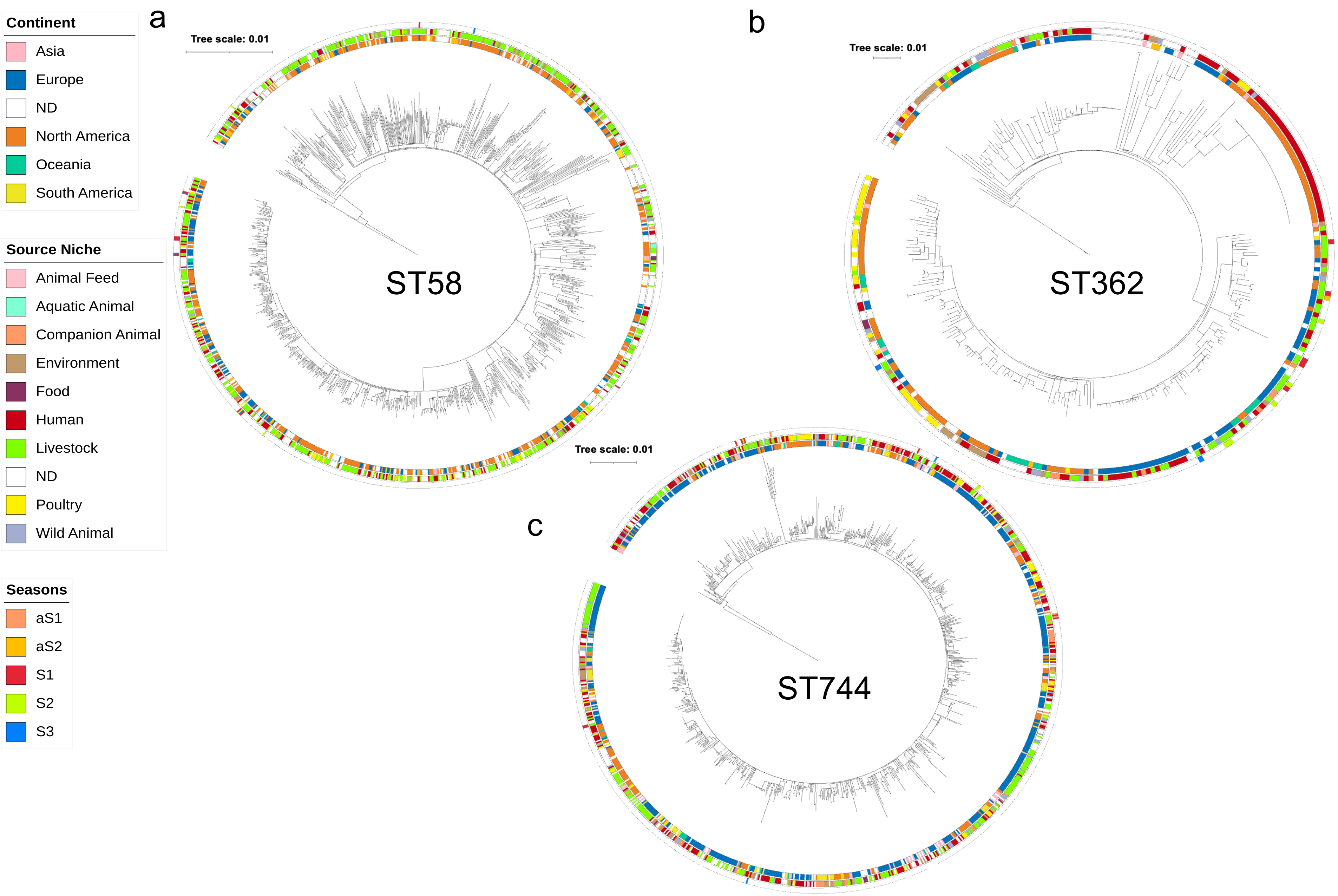

Fig. S7. Phylogeny and characteristics of ST58, ST362 and ST744 *E. coli* isolates from the 5 seasons combined with EnteroBase isolates. Maximum likelihood phylogeny was performed by using RAxML 8.2.12 [5] based on recombination-free core genome alignment, computed with Snippy 4.6.0 and Gubbins 2.4.1 to filter recombined regions[6]. **a.** ST58 2730 EnteroBase strains (2330 annotated), 360 of human and 536 of bovine origin. **b**, ST362, 310 EnteroBase isolates (248 annotated), 100 of human and 34 of bovine origin. **c**, ST744, 1012 EnteroBase isolates (831 annotated), 260 of human and 49 of bovine origin. Genome were annotated according to the figure key as circles from inside to outside : the continent of origin, the source niche, and the season of isolation. No cluster was identified in these three STs.

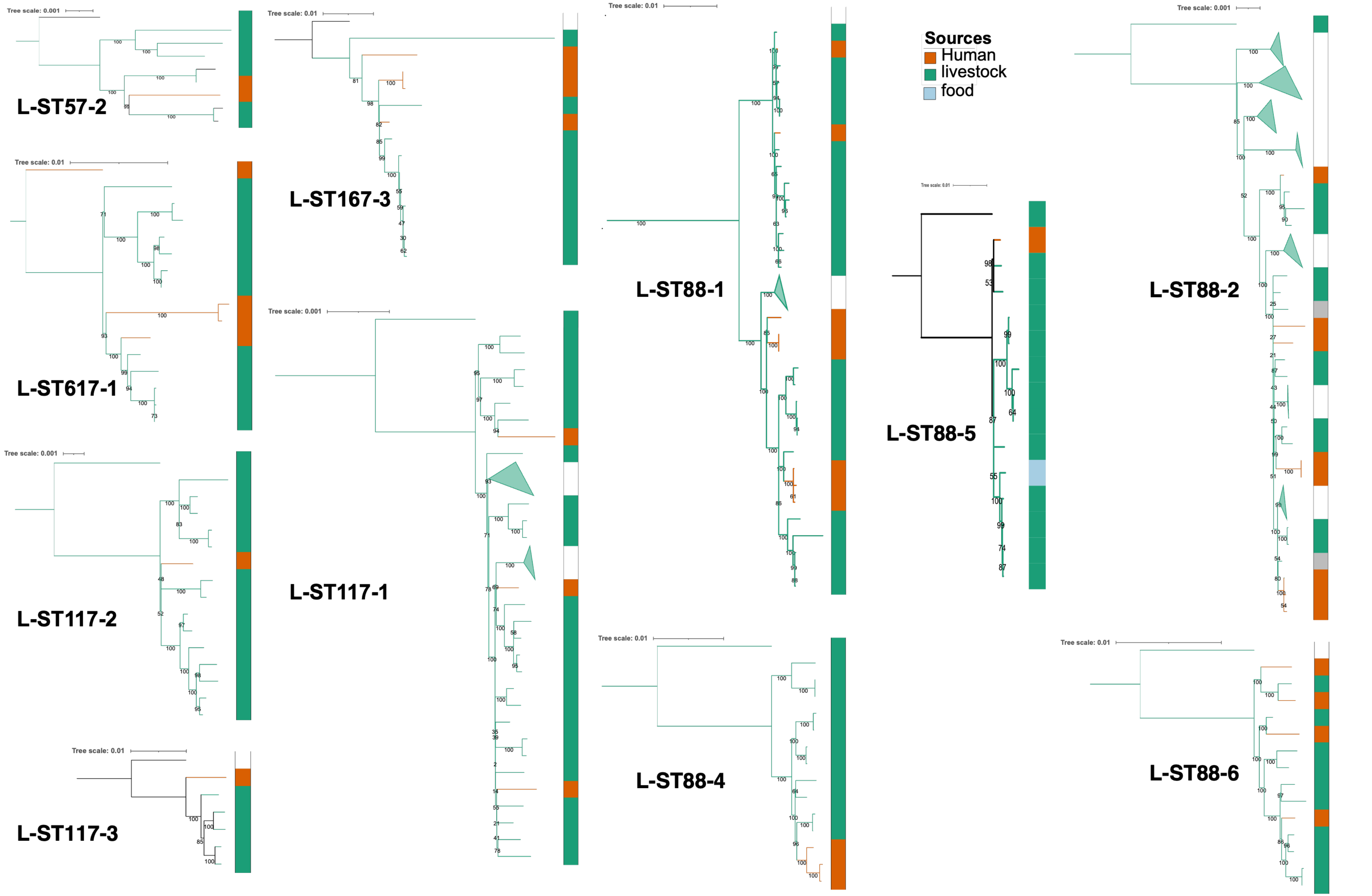

Fig. S8. Ancestral host of the 11 lineages including EnteroBase isolates of human origin. Phylogenetic trees and PastML[8] predictions were performed on all the isolates from an ST and sub-trees from each lineage were extracted. Host probability of the ancestral host are indicated in green, bovine more likely, or in red, human more likely. Bovin isolates are in green and human isolates in red. Bootstrap values are in black. Details on lineages are in Table 2.

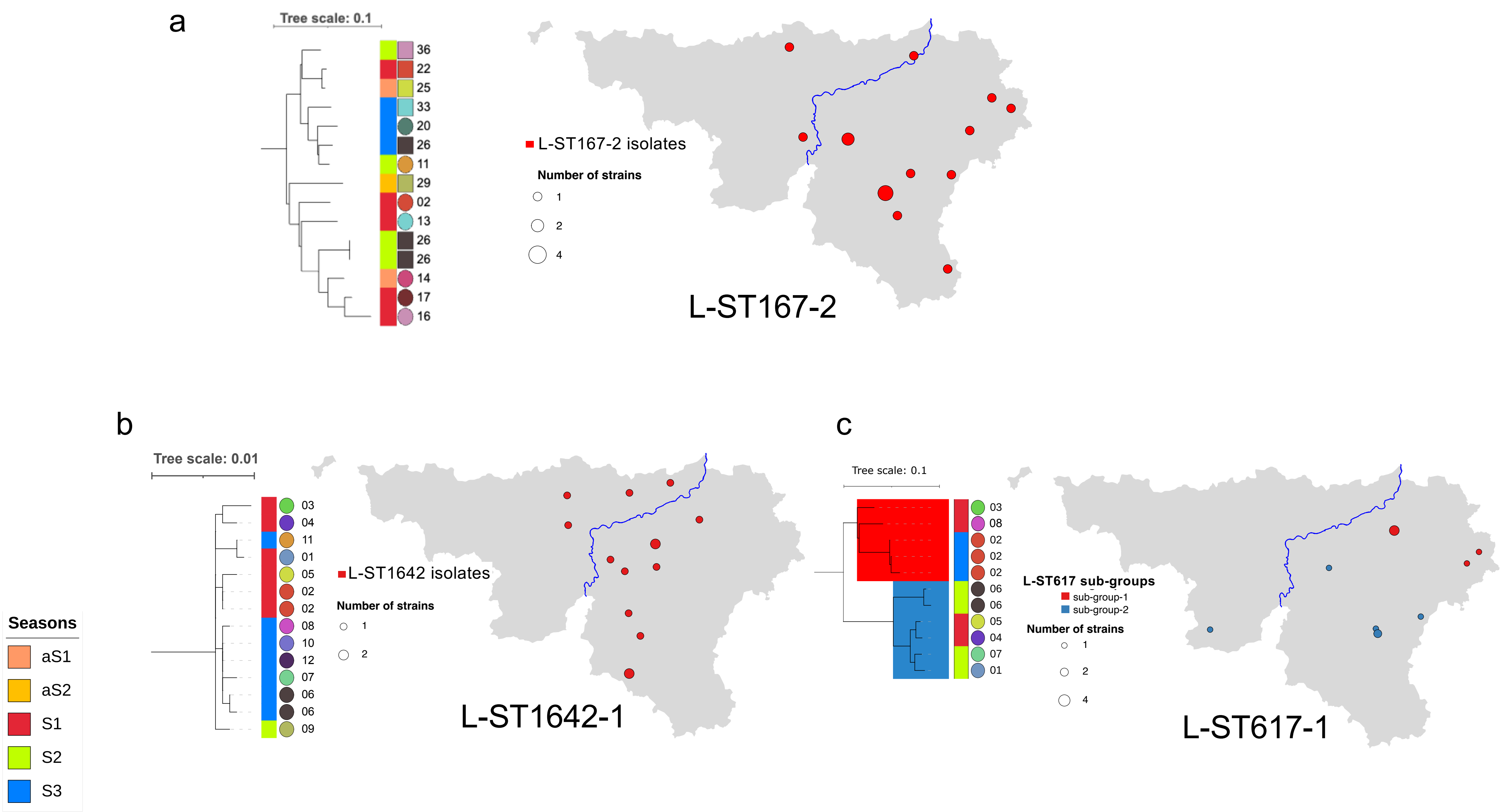

Fig. S9. Local dissemination of closely related isolates and geographical specificity. The geographical localization was compared to the phylogenetic proximity by localizing isolates according to the GPS coordinates of the postal code. Disk diameters correspond to the number of isolates. Colors correspond to the branch on the tree. Isolates were annotated on the right of the tree according to the figure key. From left to right, the season (in white, EnteroBase isolates) and the herd. The blue line represents the Meuse. **a.** L-ST167-2, isolates were mainly on the east. **b.** L-ST1642-1 **C.** L-ST617-1

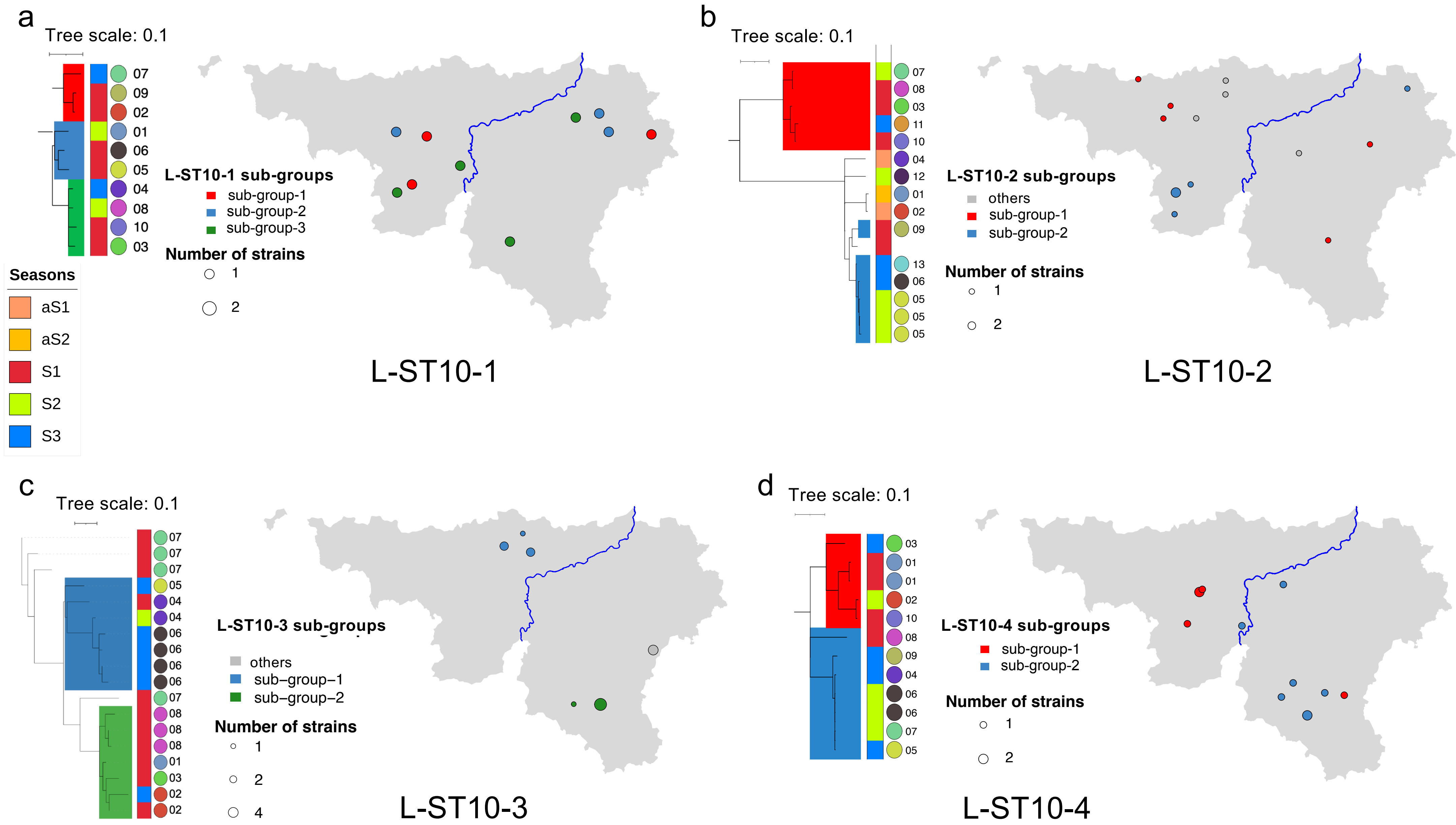

Fig. S10. Local dissemination of closely related isolates and geographical specificity. The geographical localization was compared to the phylogenetic proximity by localizing isolates according to the GPS coordinates of the postal code. Disk diameters correspond to the number of isolates. Colors correspond to the branch on the tree. Isolates were annotated on the right of the tree according to the figure key. From left to right, the season (in white, EnteroBase strains) and the herd. The blue line represents the Meuse. **a.** L-ST10-1. **b.** L-ST10-2 **c.** Lineage L-ST10-3. **d.** Lineage L-ST10-4

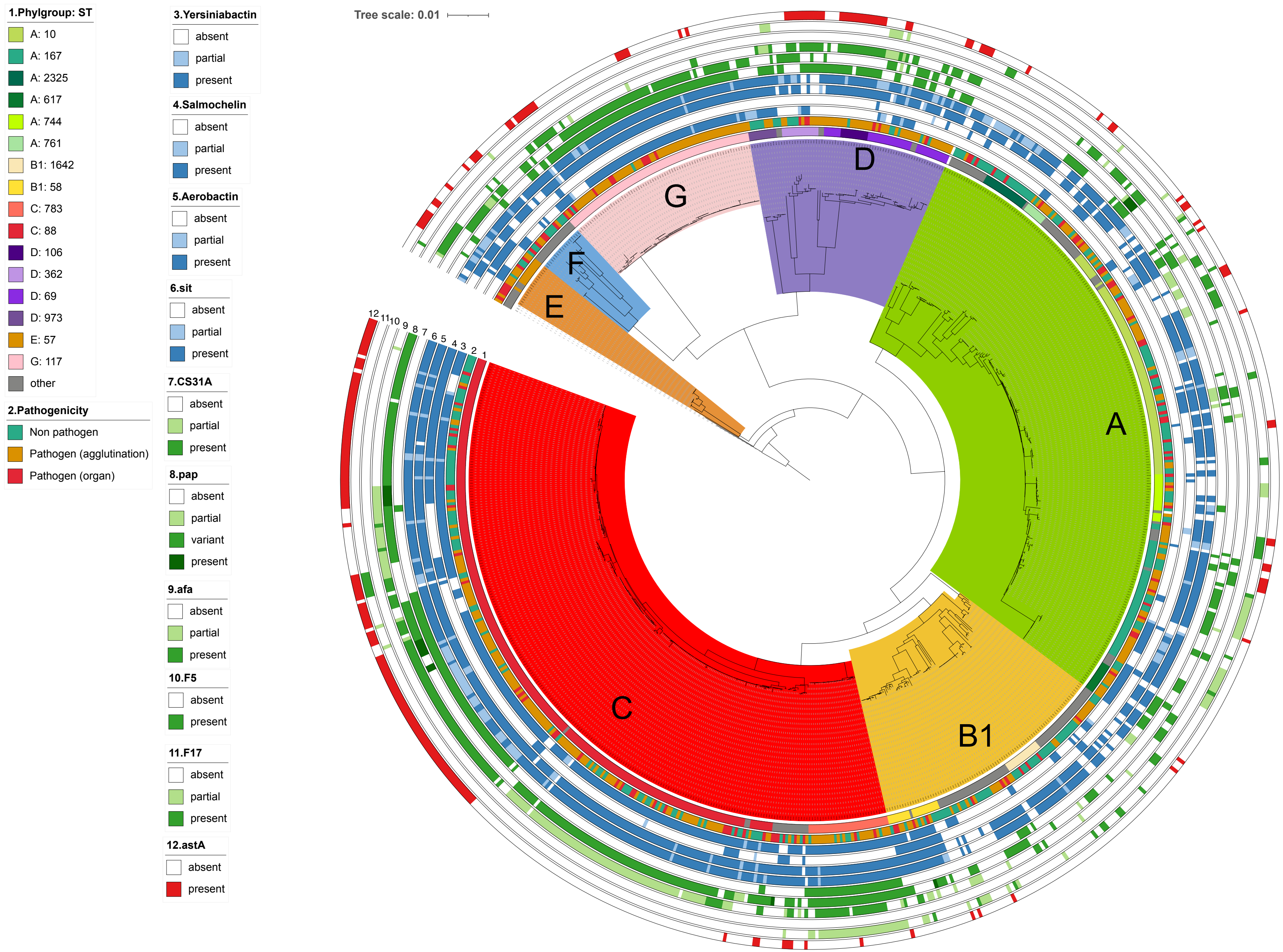

Fig. S11. Phylogeny and virulence factors of the 764 *E. coli* isolates from calves. Phylogeny was performed by maximum likelihood by using Roary, 3.13.0 and RAxML 8.2.12 [5], with *Escherichia fergusonii* (NZ_CP057657) as outgroup. Genomes were annotated according to the figure key as circles from inside to outside: the main ST and the corresponding phylogroup, the pathogenicity status and virulence factors: yersiniabactin, salmochelin, aerobactin, sit, CS31A pilus, Pap pilus, Afa pilus, F5 pilus, F17 and toxin AstA. Details on virulence factors are in Table S8. The seven phylogroups are indicated a colored sectors.

References

1 de Graaf, F. K., Krenn, B. E. & Klaasen, P. Organization and expression of genes involved in the biosynthesis of K99 fimbriae. *Infect. Immun.* **43**, 508-514 (1984). https://doi.org:10.1128/iai.43.2.508-514.1984

2 Martin, C., Boeuf, C. & Bousquet, F. *Escherichia coli* CS31A fimbriae: molecular cloning, expression and homology with the K88 determinant. *Microbial pathogenesis* **10**, 429-442 (1991). https://doi.org:10.1016/0882-4010(91)90108-m

3 Bihannic, M. *et al.* Identification and detection of three new F17 fimbrial variants in *Escherichia coli* strains isolated from cattle. *Vet Res* **45**, 76 (2014). https://doi.org:10.1186/s13567-014-0076-9

4 CASFM-vet. Comité de l'antibiogramme de la Société Française de Microbiologie RECOMMANDATIONS VÉTÉRINAIRES 2023. https://www.sfm-microbiologie.org/wp-content/uploads/2023/06/CASFM_VET2023.pdf. (2023).

5 Stamatakis, A. RAxML version 8: a tool for phylogenetic analysis and post-analysis of large phylogenies. *Bioinformatics* **30**, 1312-1313 (2014). https://doi.org:10.1093/bioinformatics/btu033

6 Croucher, N. J. *et al.* Rapid phylogenetic analysis of large samples of recombinant bacterial whole genome sequences using Gubbins. *Nucleic Acids Res.* **43**, e15 (2015). https://doi.org:10.1093/nar/gku1196

7 Deatherage, D. E. & Barrick, J. E. Identification of mutations in laboratory-evolved microbes from next-generation sequencing data using breseq. *Methods Mol Biol* **1151**, 165-188 (2014). https://doi.org:10.1007/978-1-4939-0554-6_12

8 Ishikawa, S. A., Zhukova, A., Iwasaki, W. & Gascuel, O. A Fast Likelihood Method to Reconstruct and Visualize Ancestral Scenarios. *Mol Biol Evol* **36**, 2069-2085 (2019). https://doi.org:10.1093/molbev/msz131
